# Correction: Chitinase-1 inhibition attenuates metabolic dysregulation and restores homeostasis in MASH animal models

**DOI:** 10.3389/fimmu.2025.1680129

**Published:** 2025-08-15

**Authors:** Frontiers Production Office

**Affiliations:** Frontiers Media SA, Lausanne, Switzerland

**Keywords:** chitinase 1, OATD-01, MASH, fibrosis, inflammation, macrophage, metabolism, glycolysis

Supplemental materials, Supplementary File 1 and Supplementary File 2, were erroneously omitted. The files have now been published.

The original version of this article has been updated.

