# Supplementary figures and images for: Chitinase-1 inhibition attenuates metabolic dysregulation and restores homeostasis in MASH animal models
Source: Front Immunol. 2025 May 29;16:1544973. doi: 10.3389/fimmu.2025.1544973 (PMC12158736; doi:10.3389/fimmu.2025.1544973)

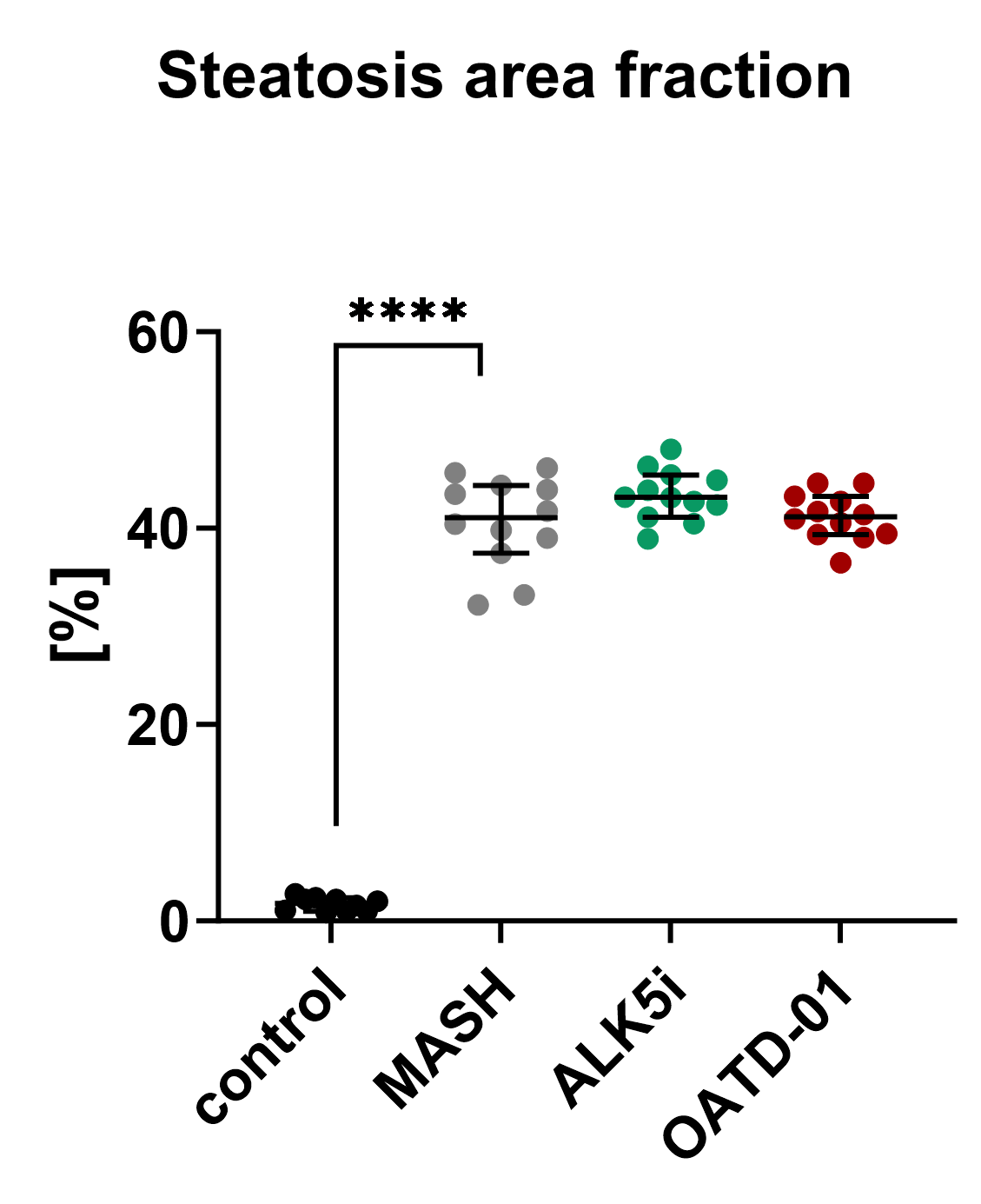

Supplement: Supplementary Figure 1 — Steatosis is induced in the MASH rat study. Analysis of steatosis in livers from experimental animals from Picro-Sirius Red stained sections (the same sections used to evaluate fibrosis: Figure 3K ) by whole slide computer-aided analysis presented as the percent of the liver section (performed with Visiopharm Image Analysis Software). Number of data points were: nControl =10, nMASH =12, nALK5i=12, nOATD-01 = 12. Statistical significance of differences between groups was evaluated separately for the Control and MASH groups using an Mann-Whitney U test. Comparisons among the MASH vs ALK5i MASH vs OATD-01 groups were conducted using one-way ANOVA. Significance was depicted as: * p < 0.05, ** p < 0.01, *** p < 0.001, **** p < 0.0001. [file Image1.tif]

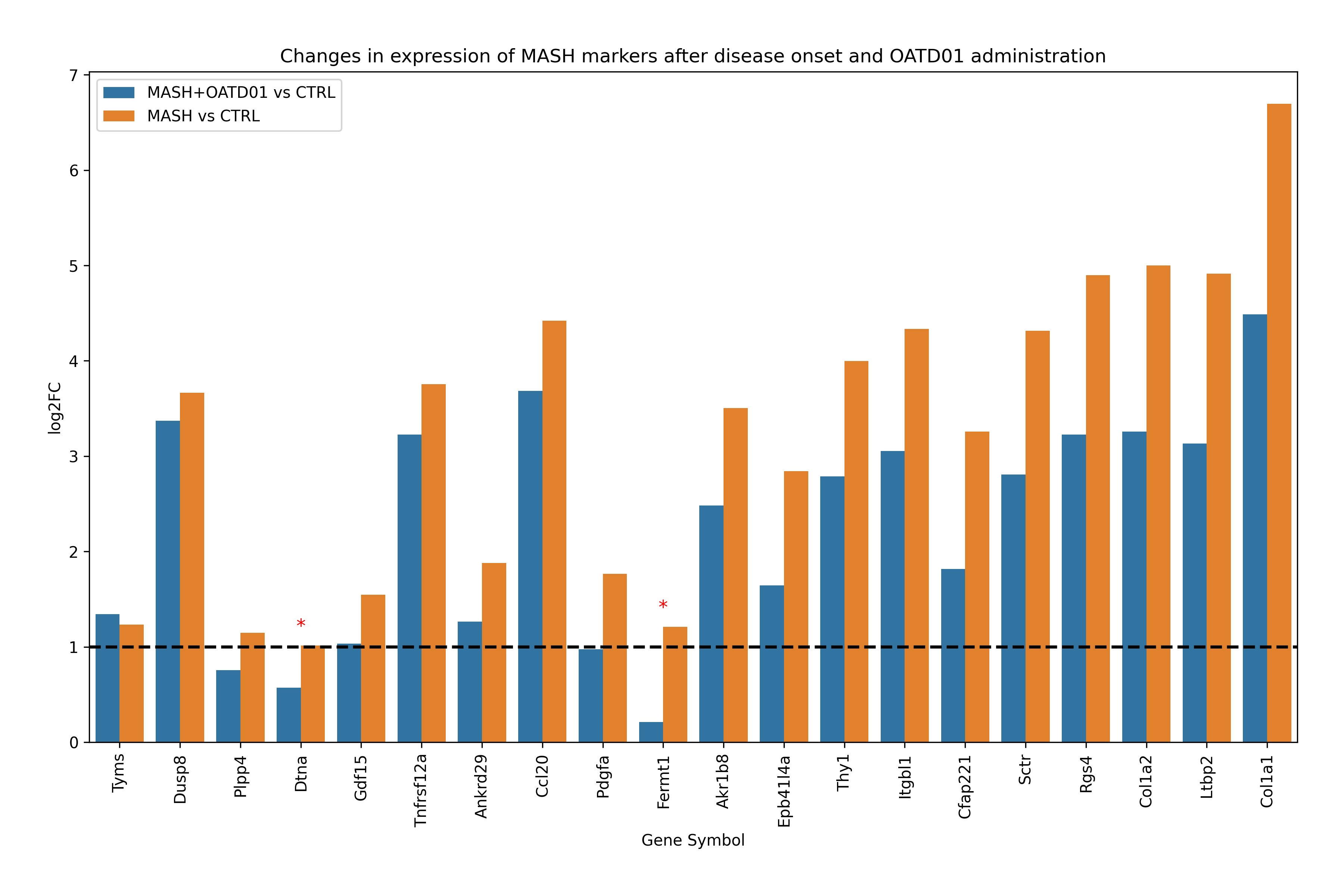

Supplement: Supplementary Figure 2 — Changes in the expression of MASH biomarkers in the MASH rat study. The changes in the form of fold change for the MASH group vs. control and MASH+OATD-01 vs. control are marked in orange and blue, respectively. The biomarkers are ranked according to fold change differences. An asterisk indicates biomarkers for which, in one or the other study, padj > 0.05. [file Image2.jpeg]

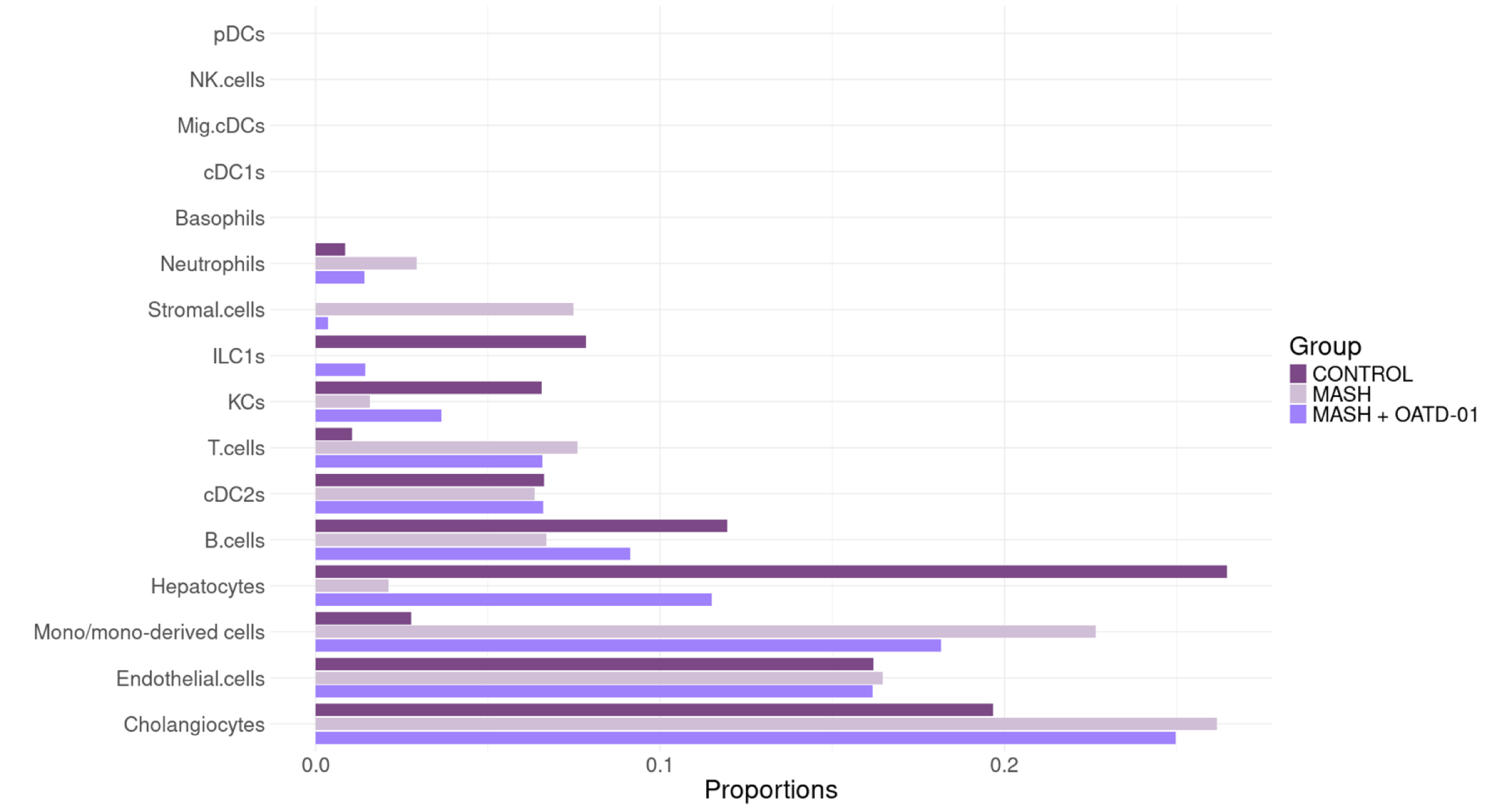

Supplement: Supplementary Figure 3 — OATD-01 restores cellular composition affected in MASH condition from the MASH rat study. Deconvolution of RNAseq dissecting proportions of different populations of cells in livers in control, MASH, and OATD-01-treated rats. [file Image3.tiff]

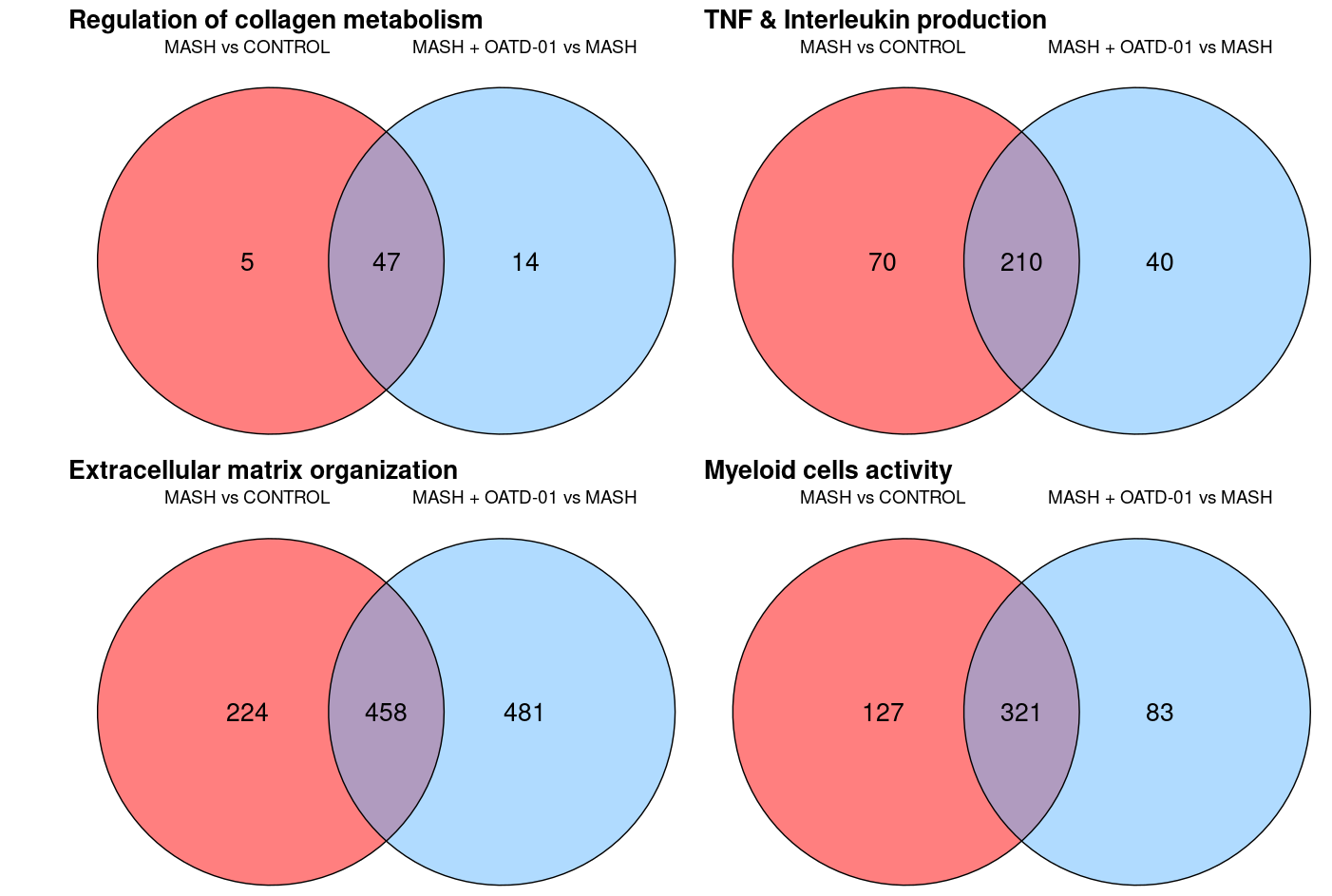

Supplement: Supplementary Figure 4 — OATD-01 reverses the expression of a significant number of genes implicated in MASH pathogenesis. Venn Diagrams of selected clustered GO processes indicated by GSEA analysis of transcriptomic data from control, MASH, and MASH + OATD-01 groups in the MASH rat study. [file Image4.tiff]

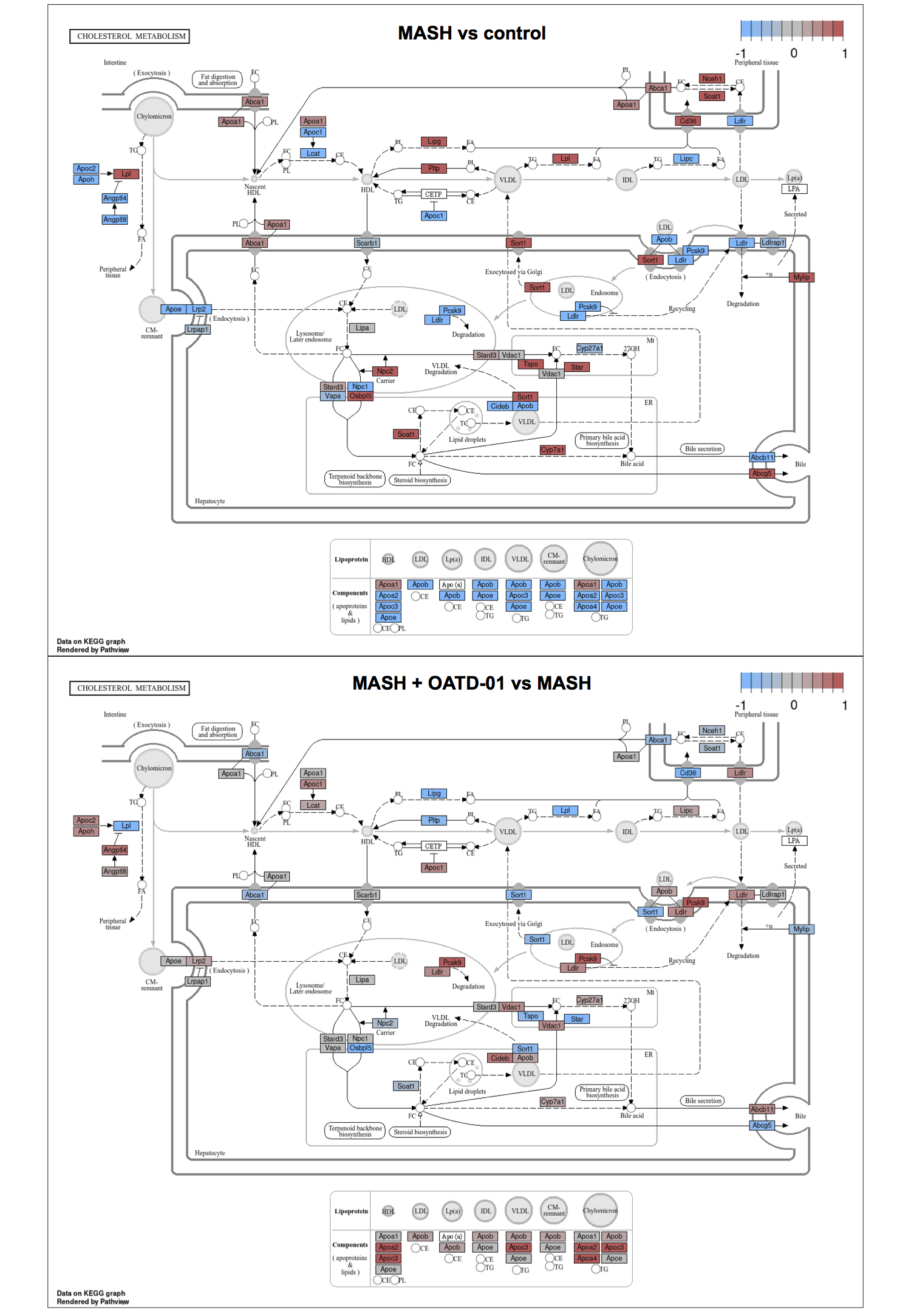

Supplement: Supplementary Figure 5 — OATD-01 regulates cholesterol flux by reversing changes in gene expression observed in MASH condition from the rat study. The cholesterol metabolism pathway from the KEGG database, with indicated genes found in the transcriptomic analysis, shows changes in gene expression for MASH vs Control and MASH + OATD-01 vs MASH. Gene expression changes are presented using log2(FoldChange) and visualized through a color gradient, where blue indicates strong downregulation and red indicates strong upregulation. [file Image5.tiff]

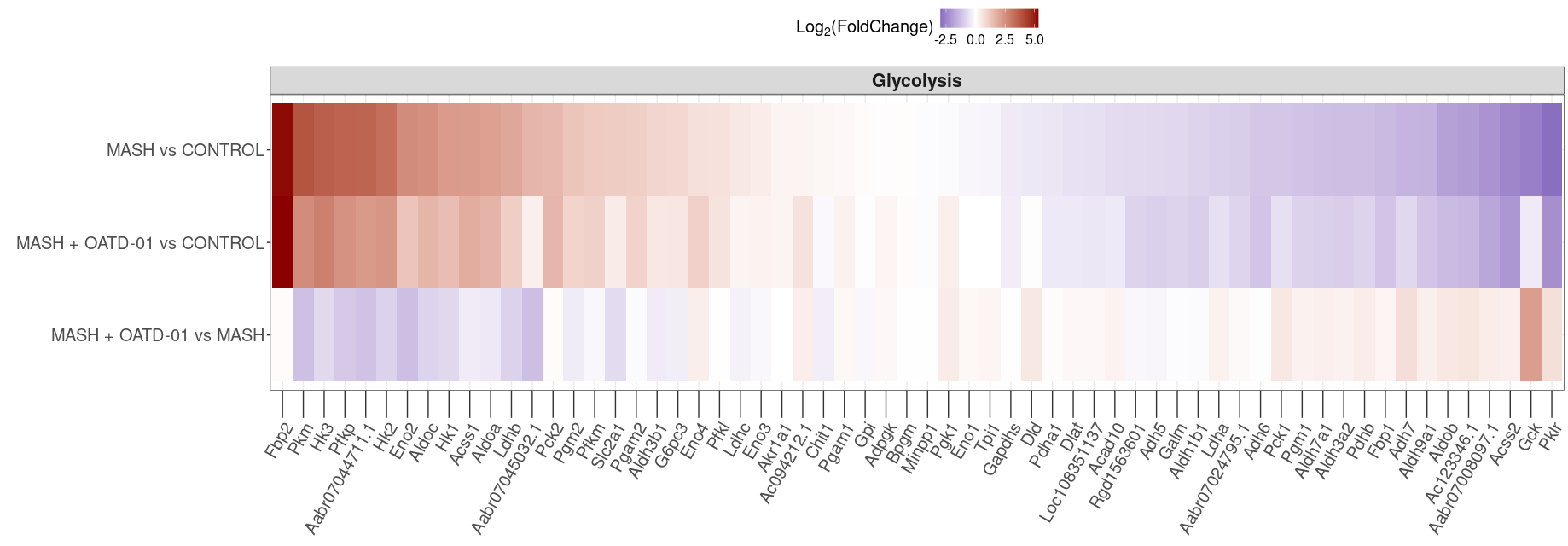

Supplement: Supplementary Figure 6 — Glycolysis is altered in MASH and regulated by OATD-01. Heatmaps displaying the expression of genes involved in glycolysis and TCA cycle from MASH rat study. Genes were fetched from the KEGG database. Gene expression changes are presented using log2(FoldChange) and visualized through a color gradient where blue indicates strong downregulation, and red indicates strong upregulation. [file Image6.tiff]

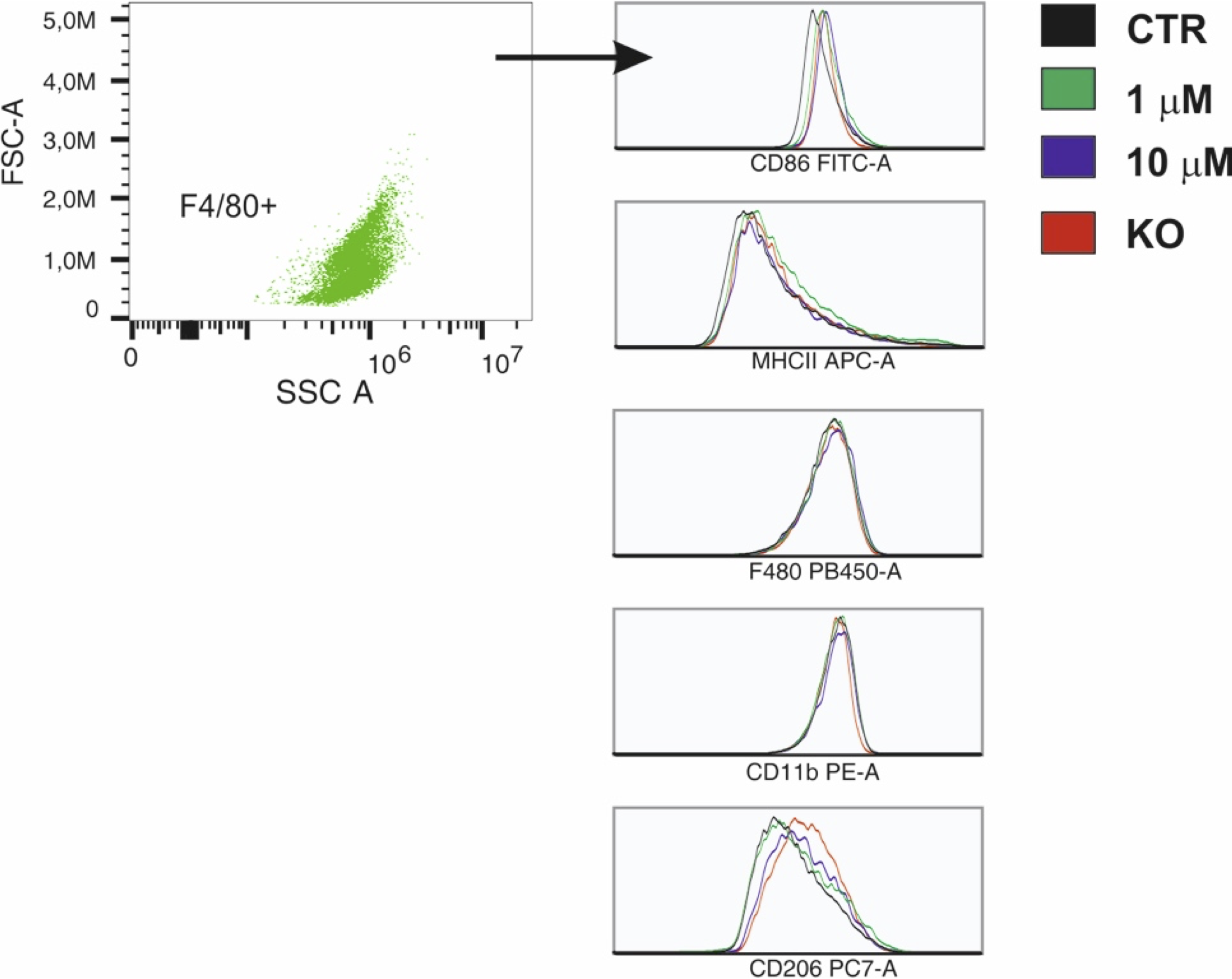

Supplement: Supplementary Figure 7 — OATD-01 and CHIT KO do not affect the phenotype of BMDMs stimulated with LPS. FACs analysis of LPS-treated BMDMs from WT and CHIT1 KO mice. [file Image7.tiff]

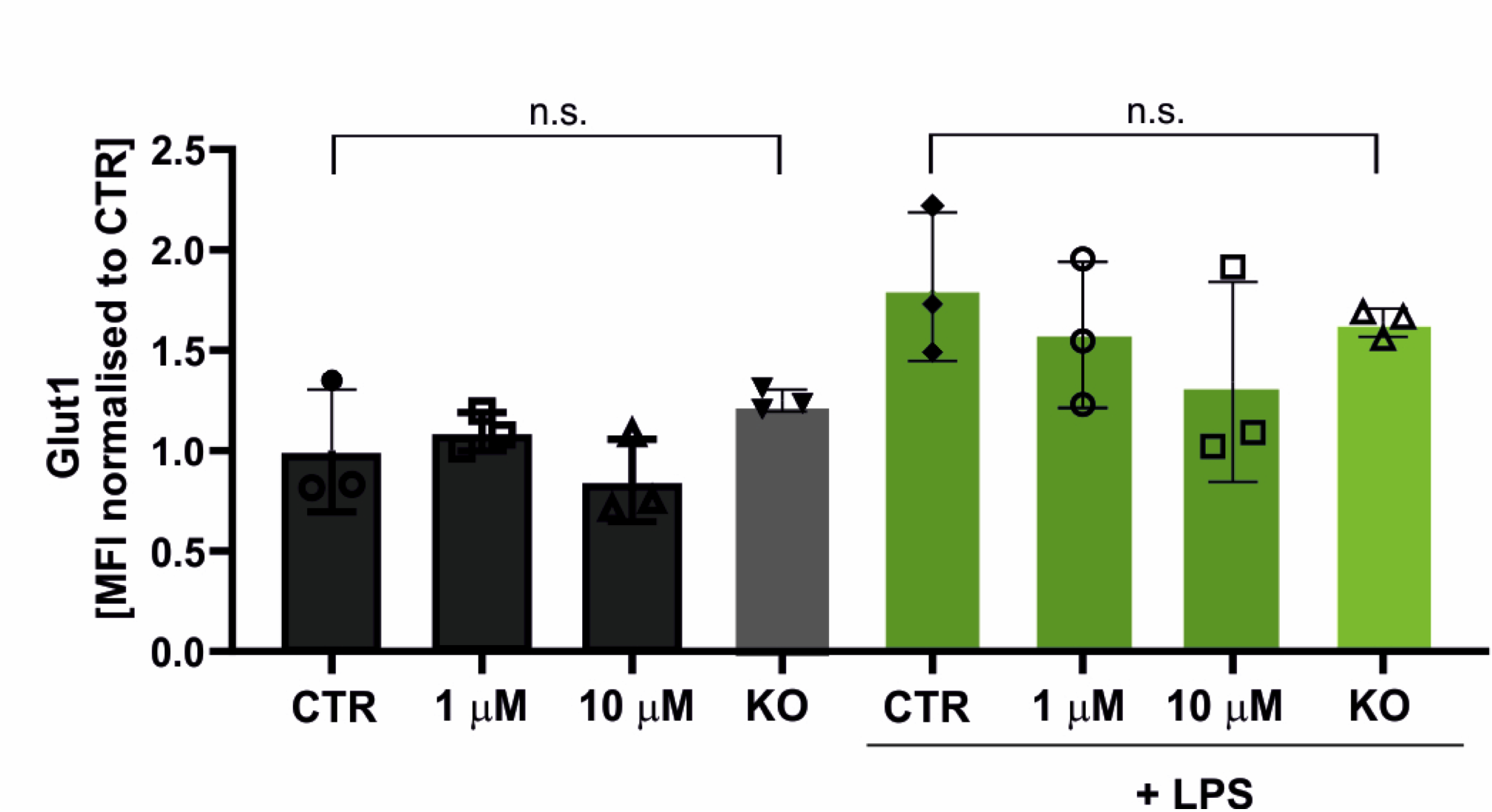

Supplement: Supplementary Figure 8 — OATD-01 and CHIT1 KO do not affect levels of GLUT1 transporter on the surface of macrophages. FACs analysis of GLUT1 extracellular domain in LPS-treated BMDMs for 48h from WT and KO mice (n=3 biological replicates). Data presented are shown as means and standard deviation. The statistical test is an unpaired t-test. Statistical significance between groups is indicated as follows: * p < 0.05, ** p < 0.01, *** p < 0.001, **** p < 0.0001. [file Image8.tiff]

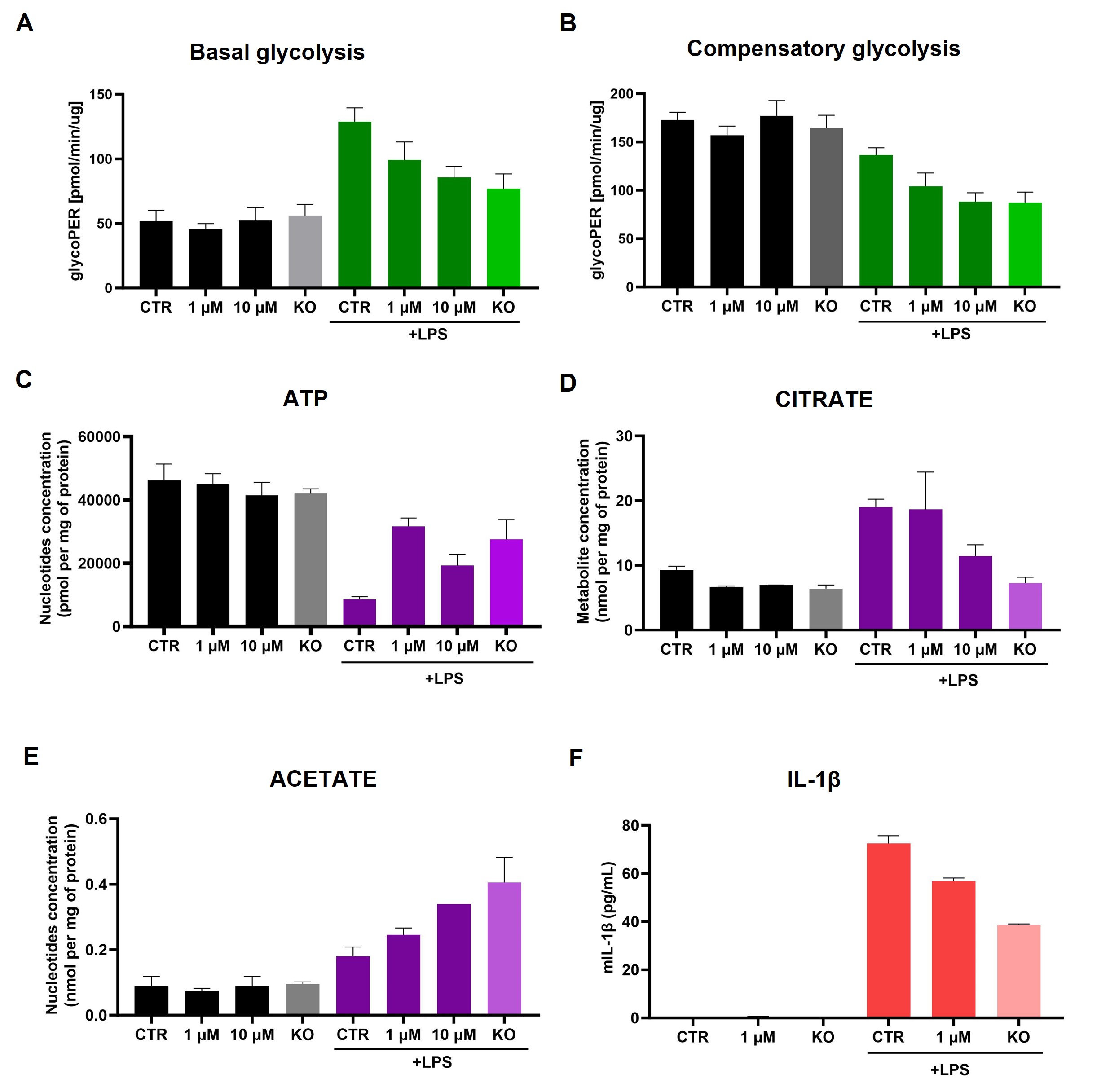

Supplement: Supplementary Figure 9 — OATD-01 regulates immunometabolism in LPS-stimulated BMDMs. (A) Measurement of basal glycolysis and (B) compensatory glycolysis (one representative biological replicate, four technical replicates per biological replicate) by Seahorse assay. Levels of metabolites: (C) ATP (n=2 biological replicates) (D) citrate (n=2 biological replicates), (E) acetate (n=2 biological replicates), and (F) levels of cytokine IL-1β (a representative experiment from n=2 biological replicates) measured in BMDMs prepared and stimulated with LPS for 48h as described in Figure 6B . Data presented are shown as means and standard deviation. [file Image9.jpeg]
