# Supplementary material for: Chitinase-1 inhibition attenuates metabolic dysregulation and restores homeostasis in MASH animal models
Source: Front Immunol. 2025 May 29;16:1544973. doi: 10.3389/fimmu.2025.1544973 (PMC12158736; doi:10.3389/fimmu.2025.1544973)

MASH vs Control up regulated clusters

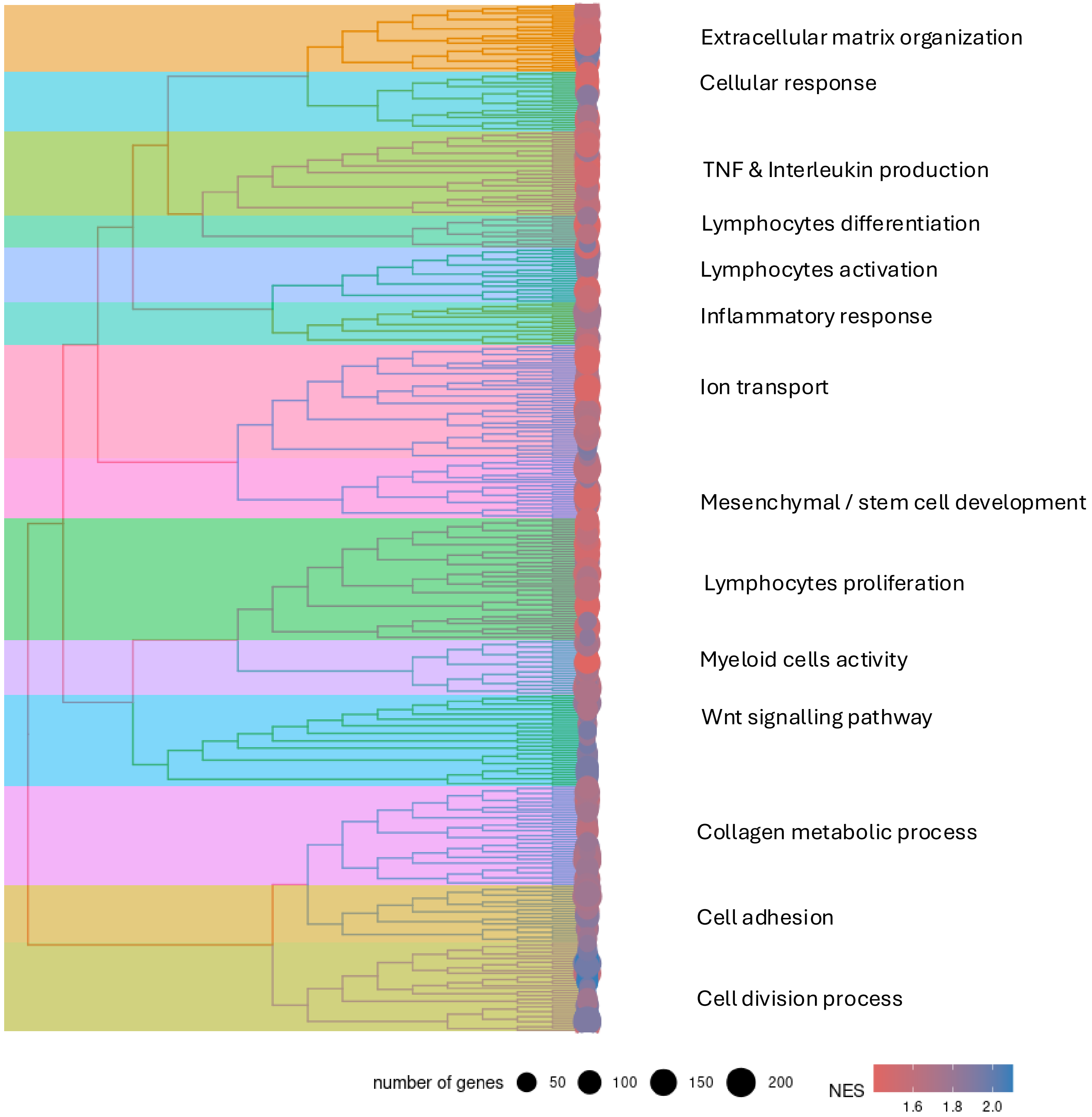

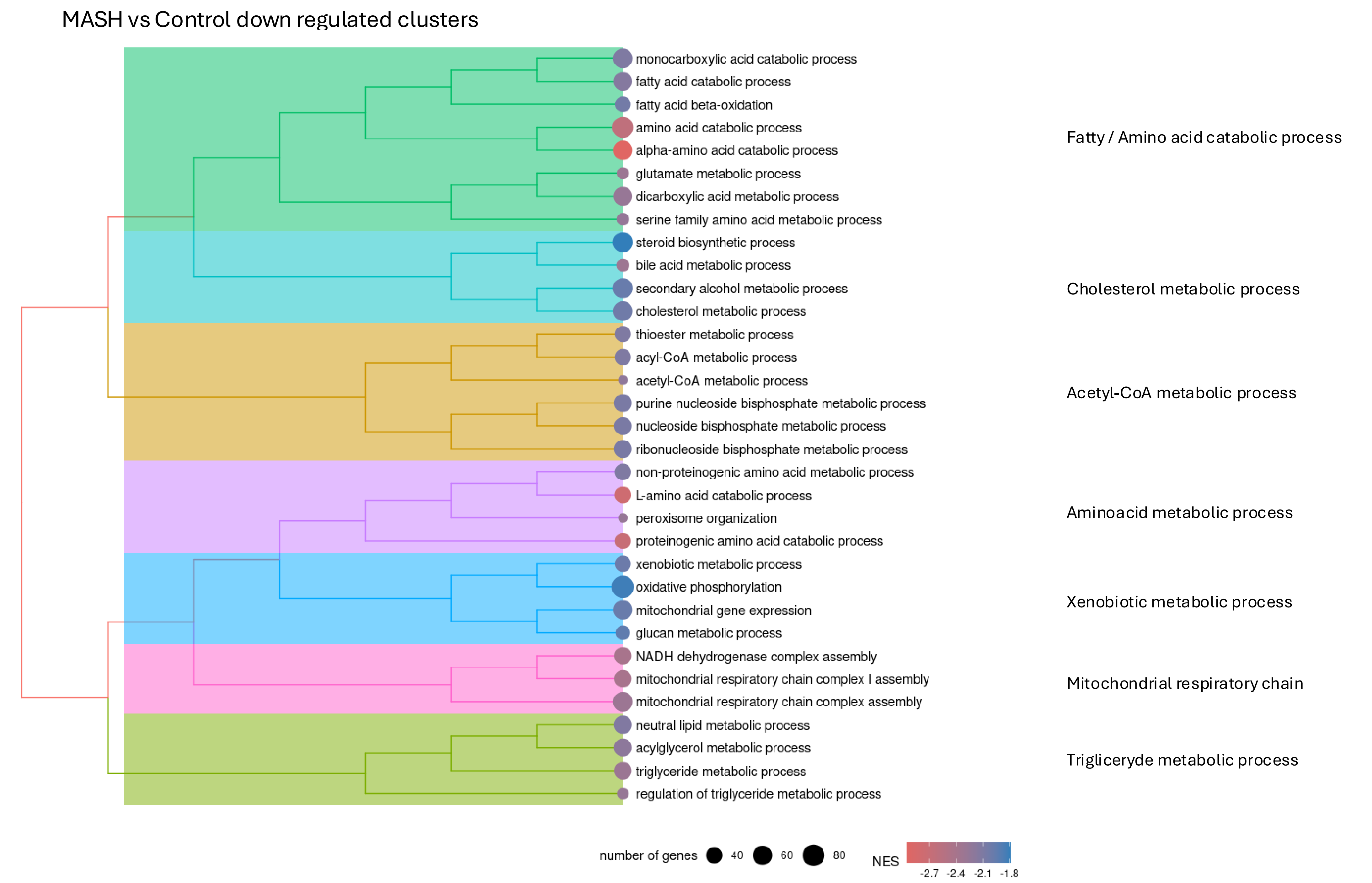
MASH vs Control down regulated clusters
monocarboxylic acid catabolic process
fatty acid catabolic process
fatty acid beta-oxidation
amino acid catabolic process
alpha-amino acid catabolic process
glutamate metabolic process
dicarboxylic acid metabolic process
serine family amino acid metabolic process
steroid biosynthetic process
bile acid metabolic process
secondary alcohol metabolic process
cholesterol metabolic process
thioester metabolic process
acyl-CoA metabolic process
acetyl-CoA metabolic process
purine nucleoside bisphosphate metabolic process
nucleoside bisphosphate metabolic process
ribonucleoside bisphosphate metabolic process
non-proteinogenic amino acid metabolic process
L-amino acid catabolic process
peroxisome organization
proteinogenic amino acid catabolic process
xenobiotic metabolic process
oxidative phosphorylation
mitochondrial gene expression
glucan metabolic process
NADH dehydrogenase complex assembly
mitochondrial respiratory chain complex I assembly
mitochondrial respiratory chain complex assembly
neutral lipid metabolic process
acylglycerol metabolic process
triglyceride metabolic process
regulation of triglyceride metabolic process
Fatty / Amino acid catabolic process
Cholesterol metabolic process
Acetyl-CoA metabolic process
Aminoacid metabolic process
Xenobiotic metabolic process
Mitochondrial respiratory chain
Trigliceryde metabolic process
number of genes
40
60
80
NES
-2.7 -2.4 -2.1 -1.8

MASH vs MASH + OATD-01 up regulated clusters

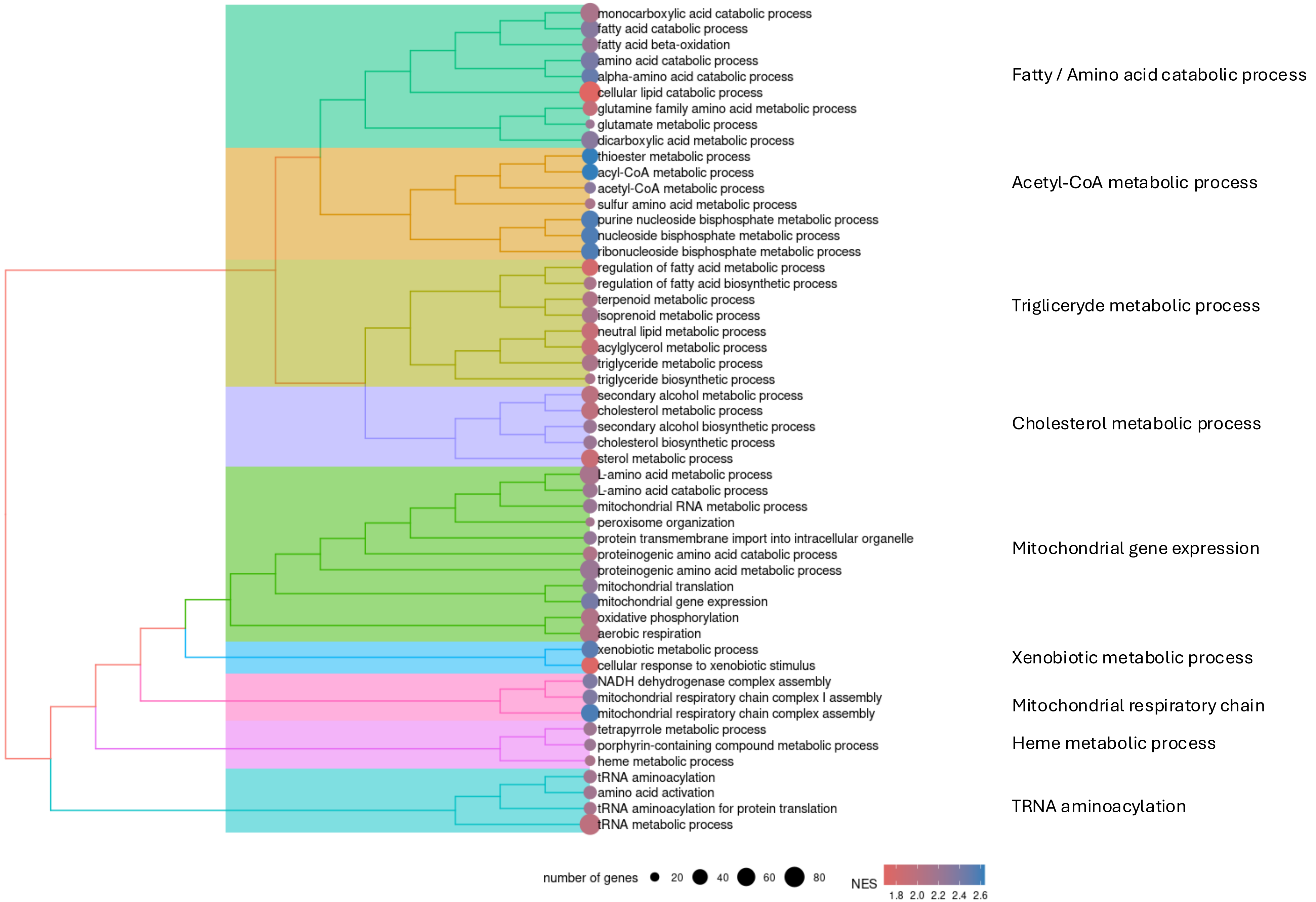

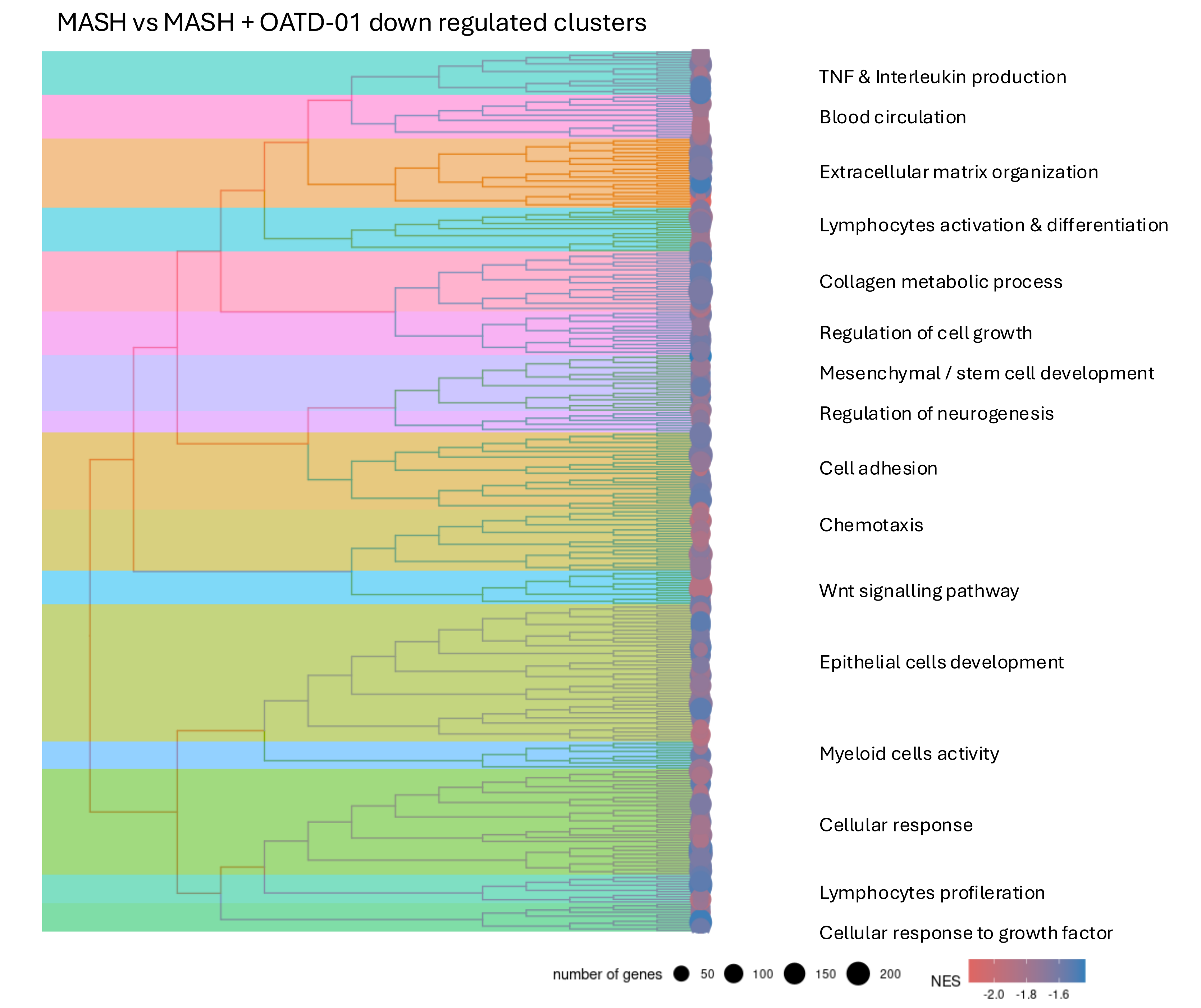
MASH vs MASH + OATD-01 down regulated clusters
TNF & Interleukin production
Blood circulation
Extracellular matrix organization
Lymphocytes activation & differentiation
Collagen metabolic process
Regulation of cell growth
Mesenchymal / stem cell development
Regulation of neurogenesis
Cell adhesion
Chemotaxis
Wnt signalling pathway
Epithelial cells development
Myeloid cells activity
Cellular response
Lymphocytes profileration
Cellular response to growth factor
number of genes
50
100
150
200
NES
-2.0
-1.8
-1.6

MASH vs Control - Extracellular matrix organization cluster

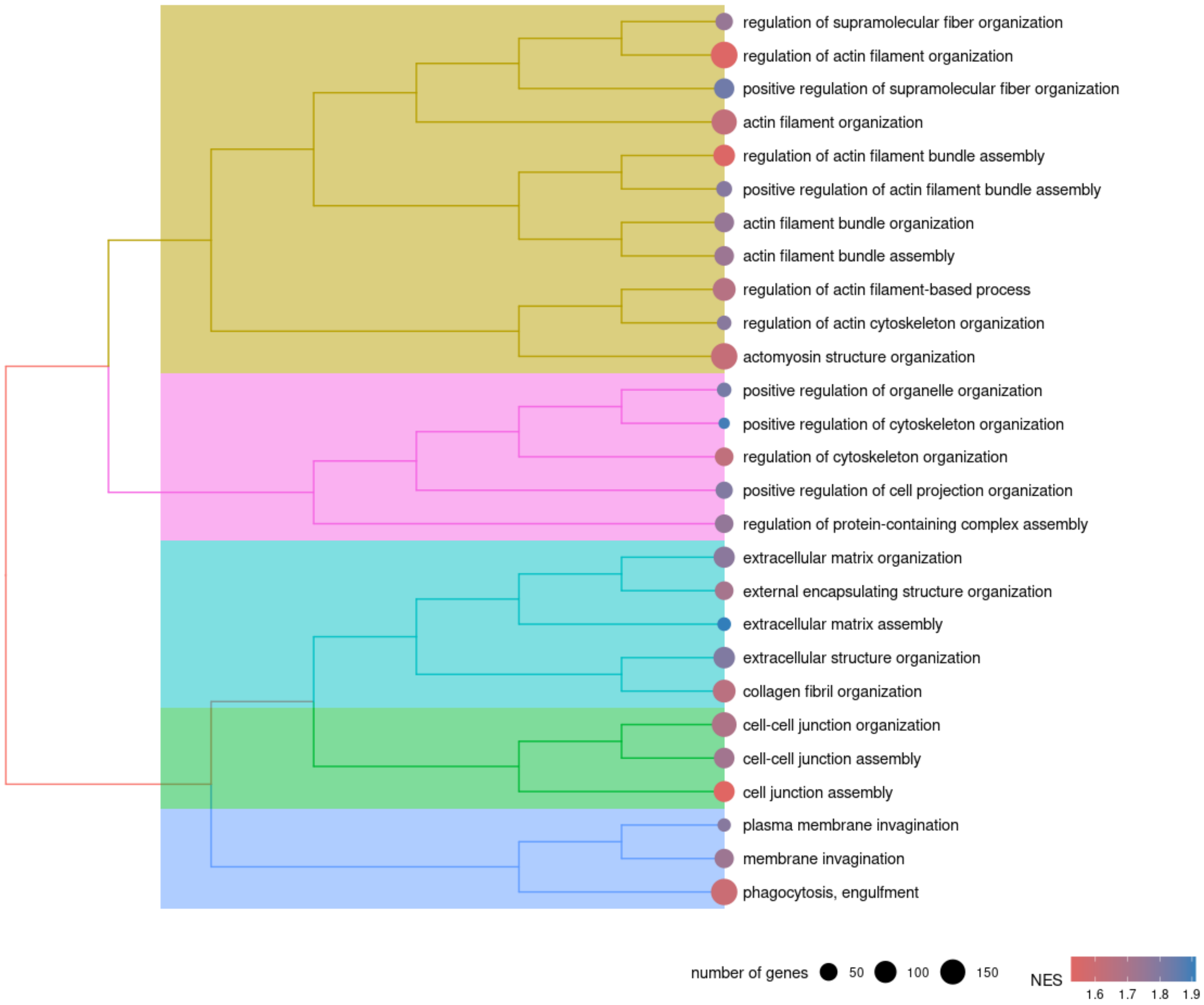

MASH vs Control Inflammatory response cluster

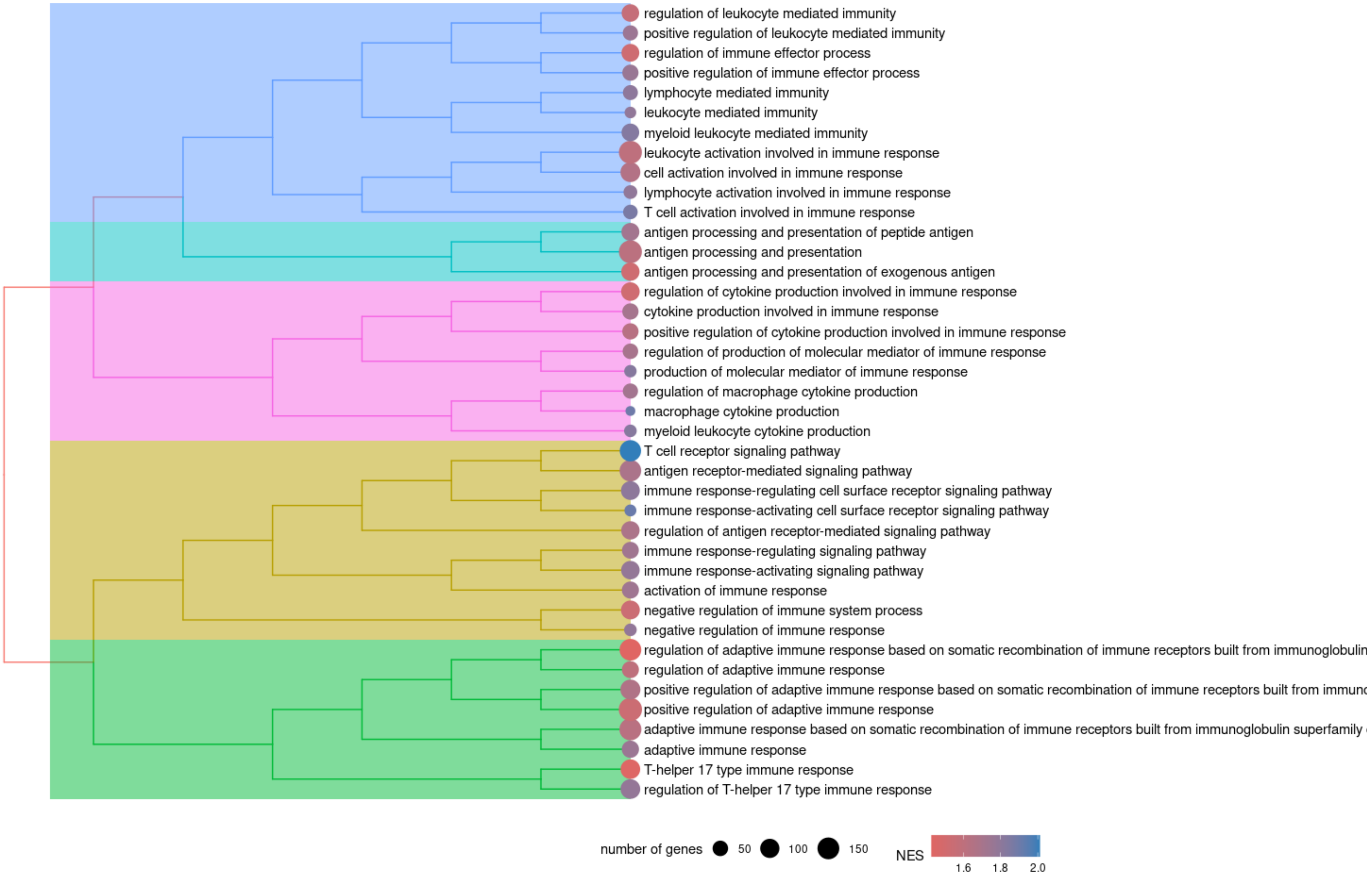

MASH vs Control Cellular response - cluster

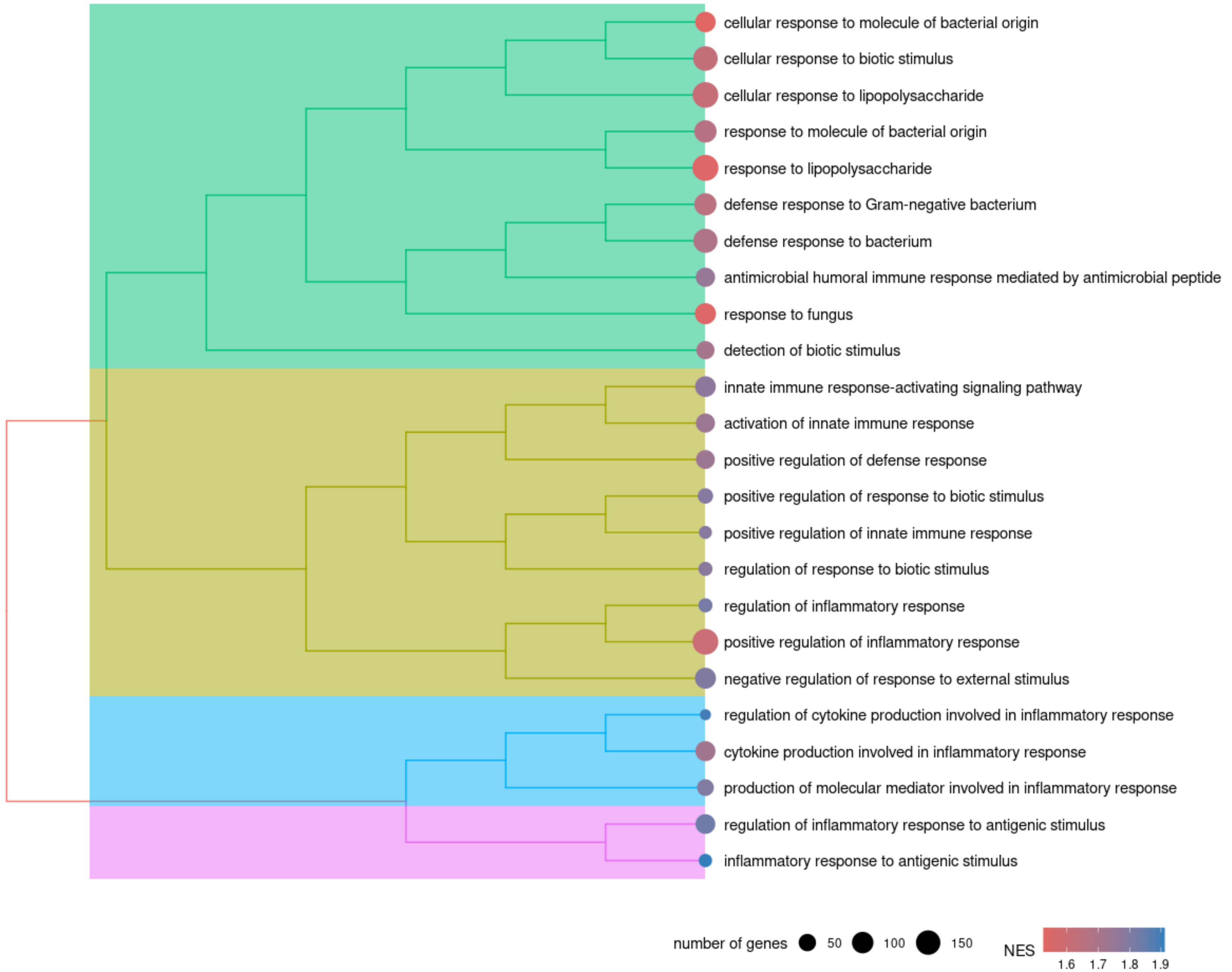

MASH vs Control - Wnt signalling pathway cluster

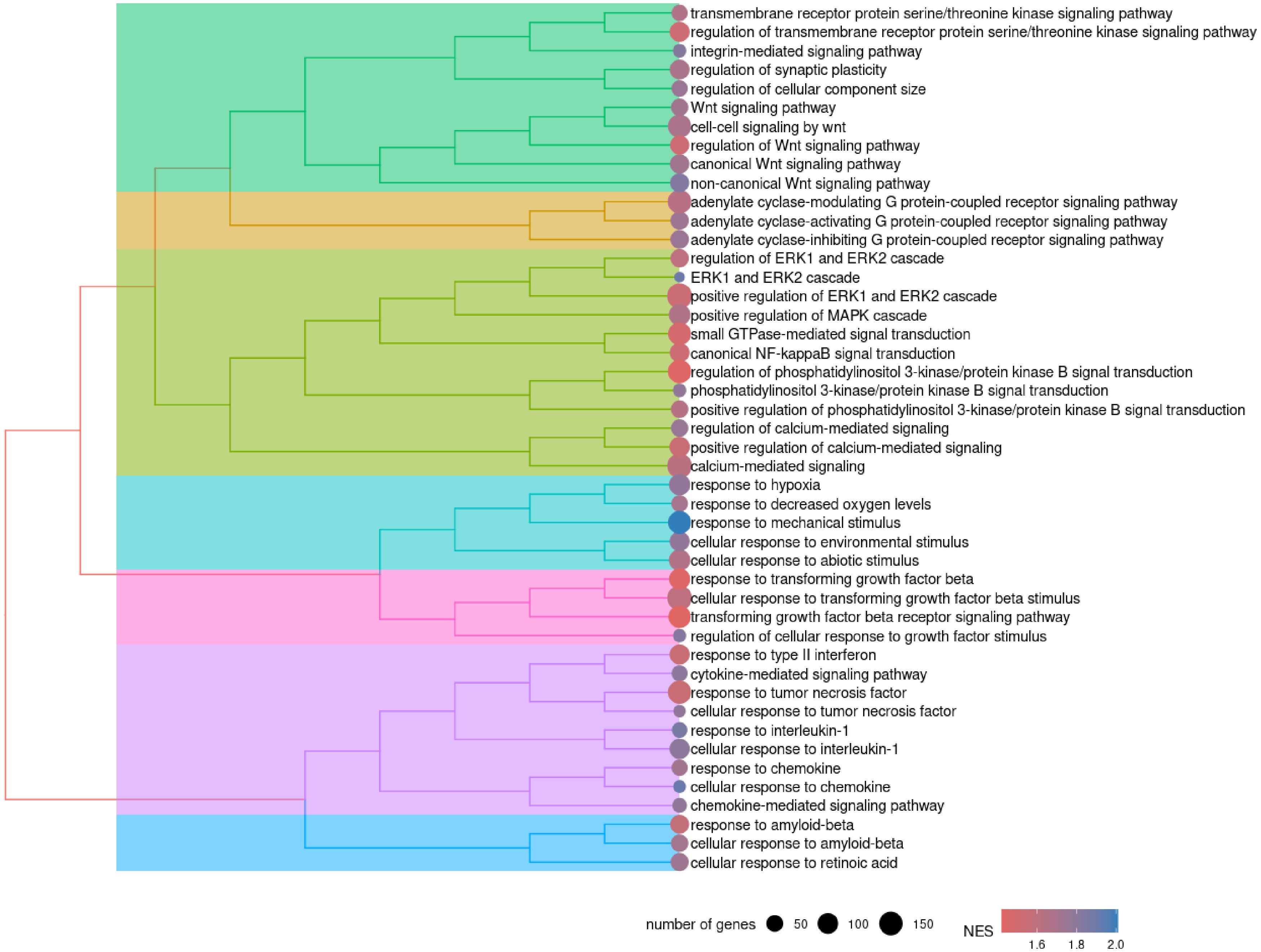

MASH vs Control - Lymphocytes activation cluster

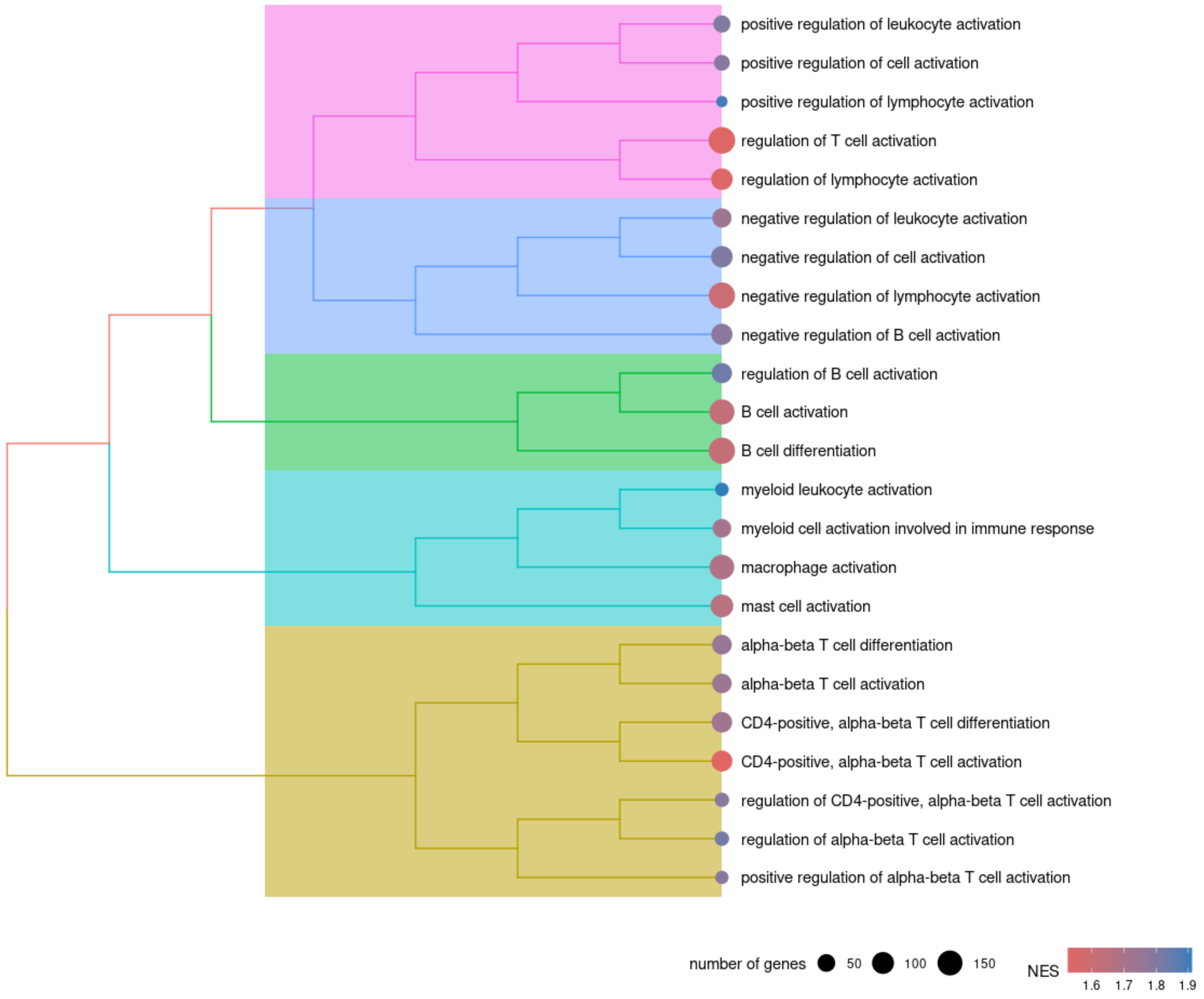

MASH vs Control - Myeloid cells activity cluster

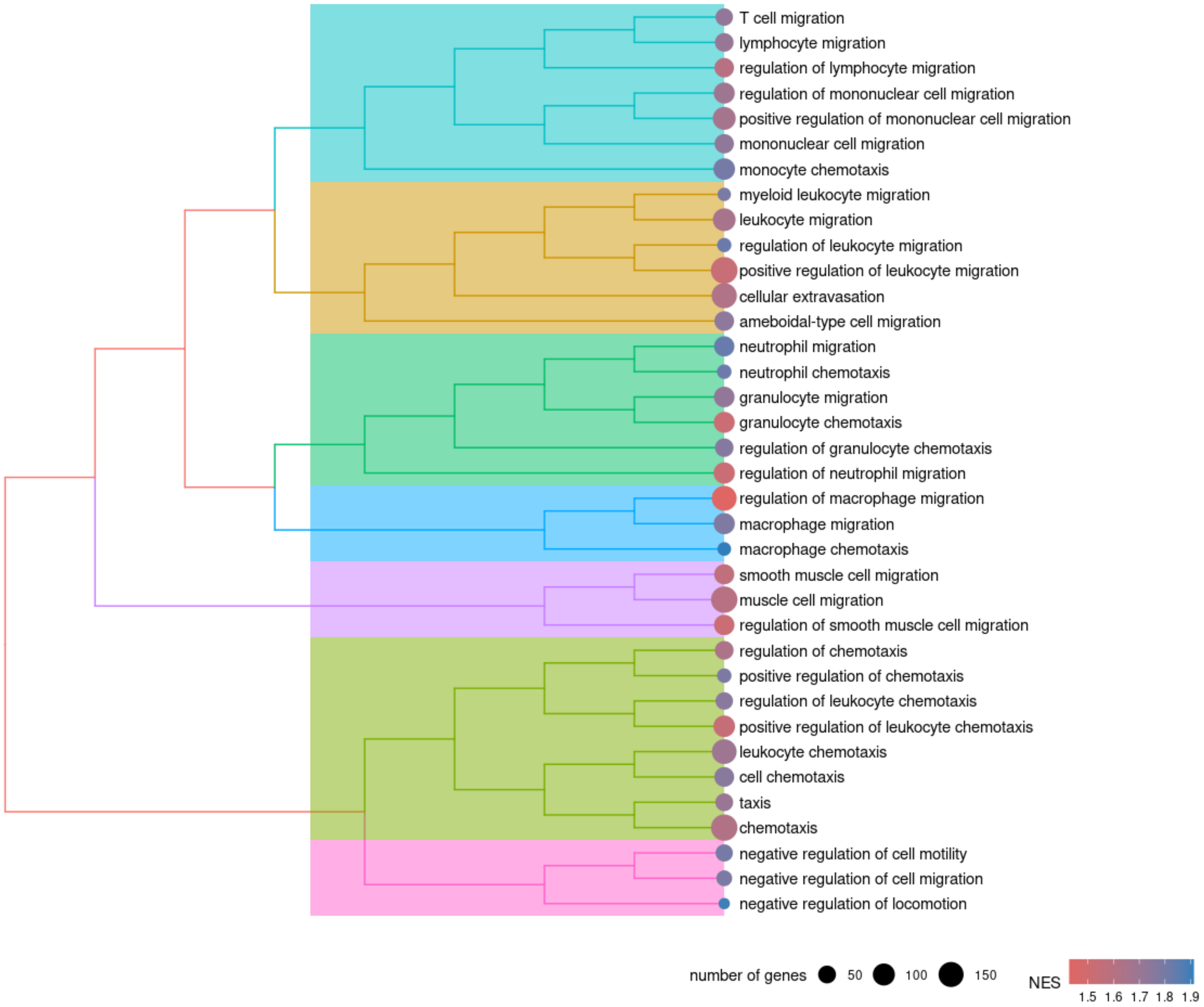

MASH vs Control - Regulation of collagen metabolism cluster

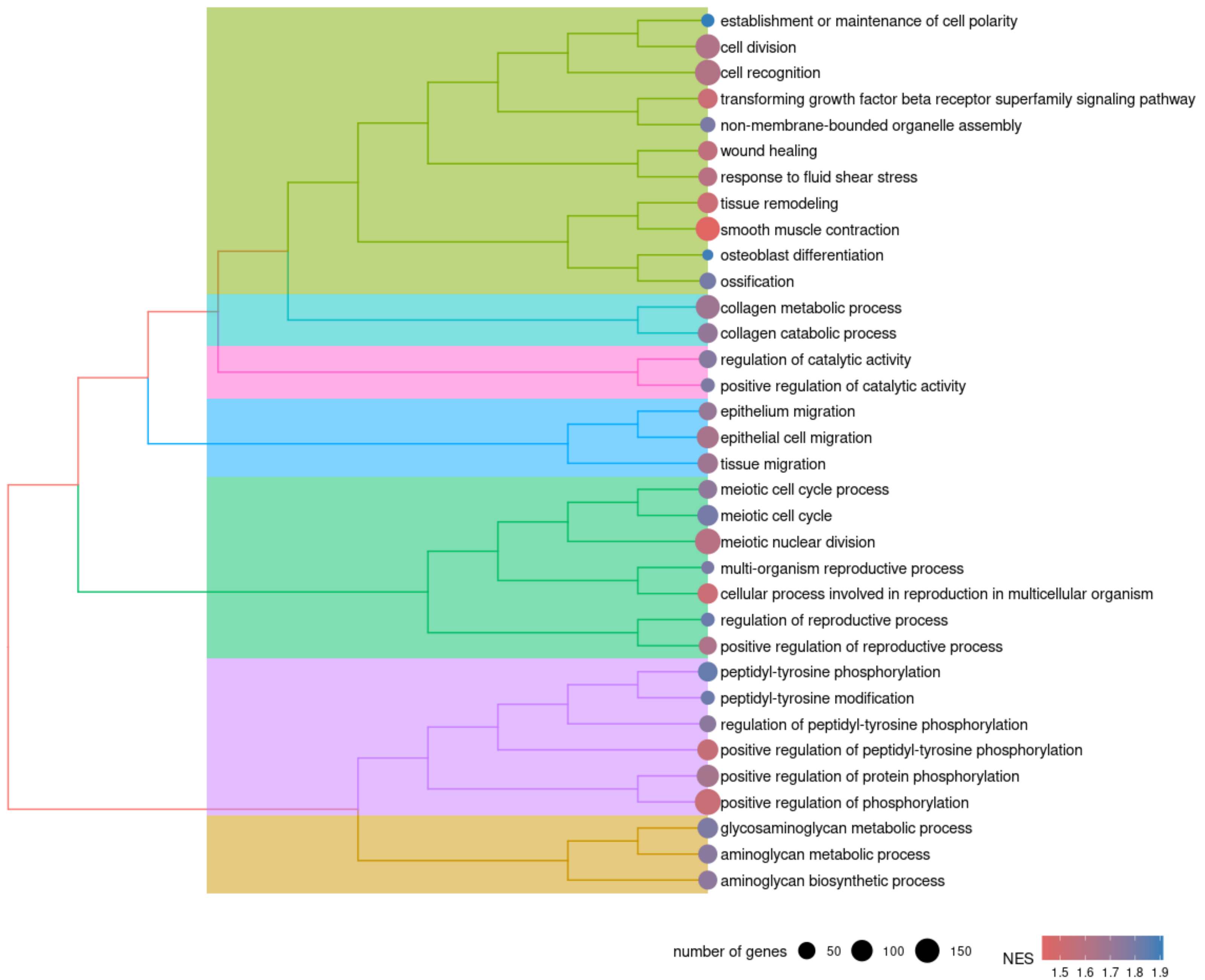

MASH vs Control - Mesenchymal / stem cell development cluster

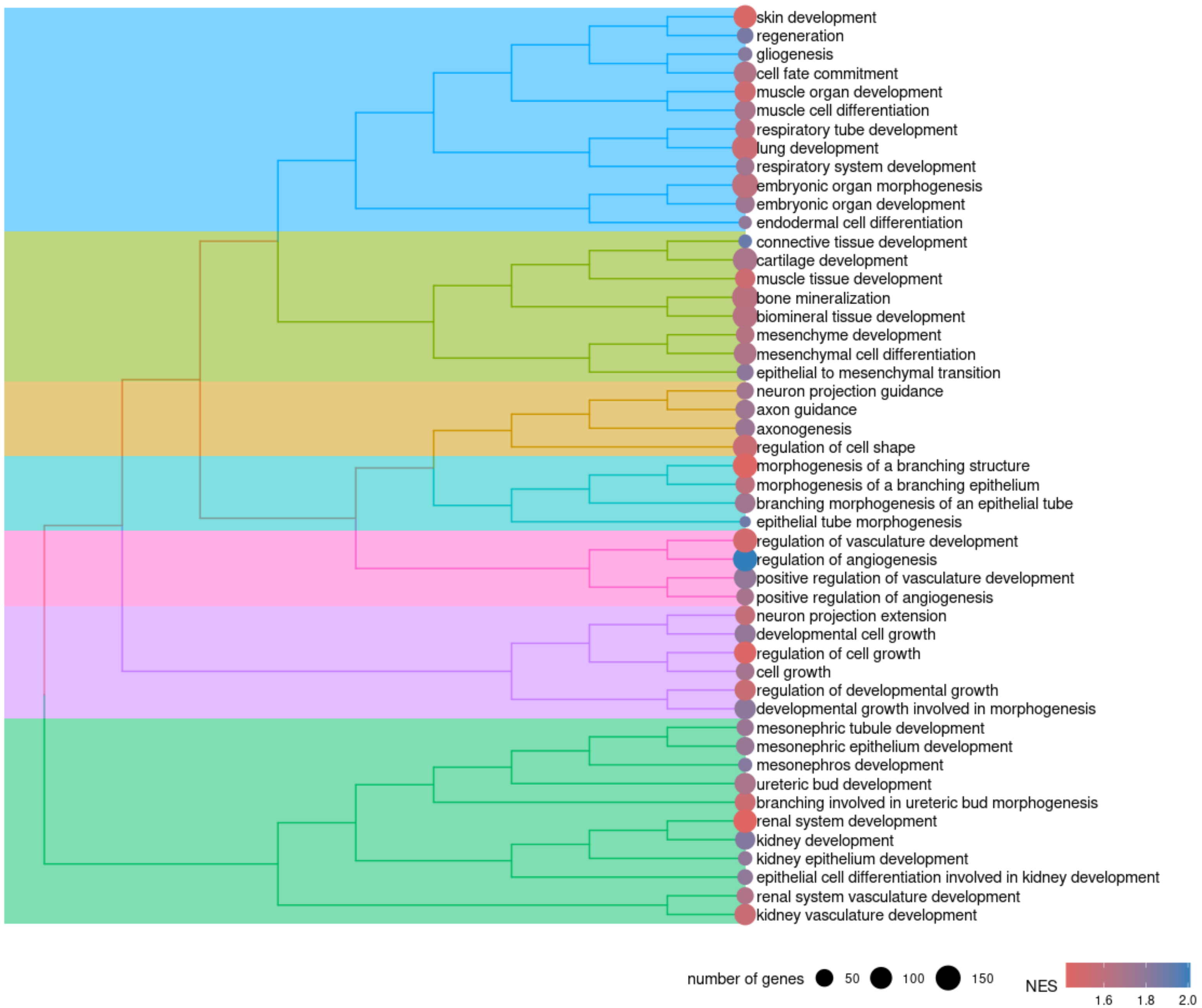

MASH vs Control - Ion transport cluster

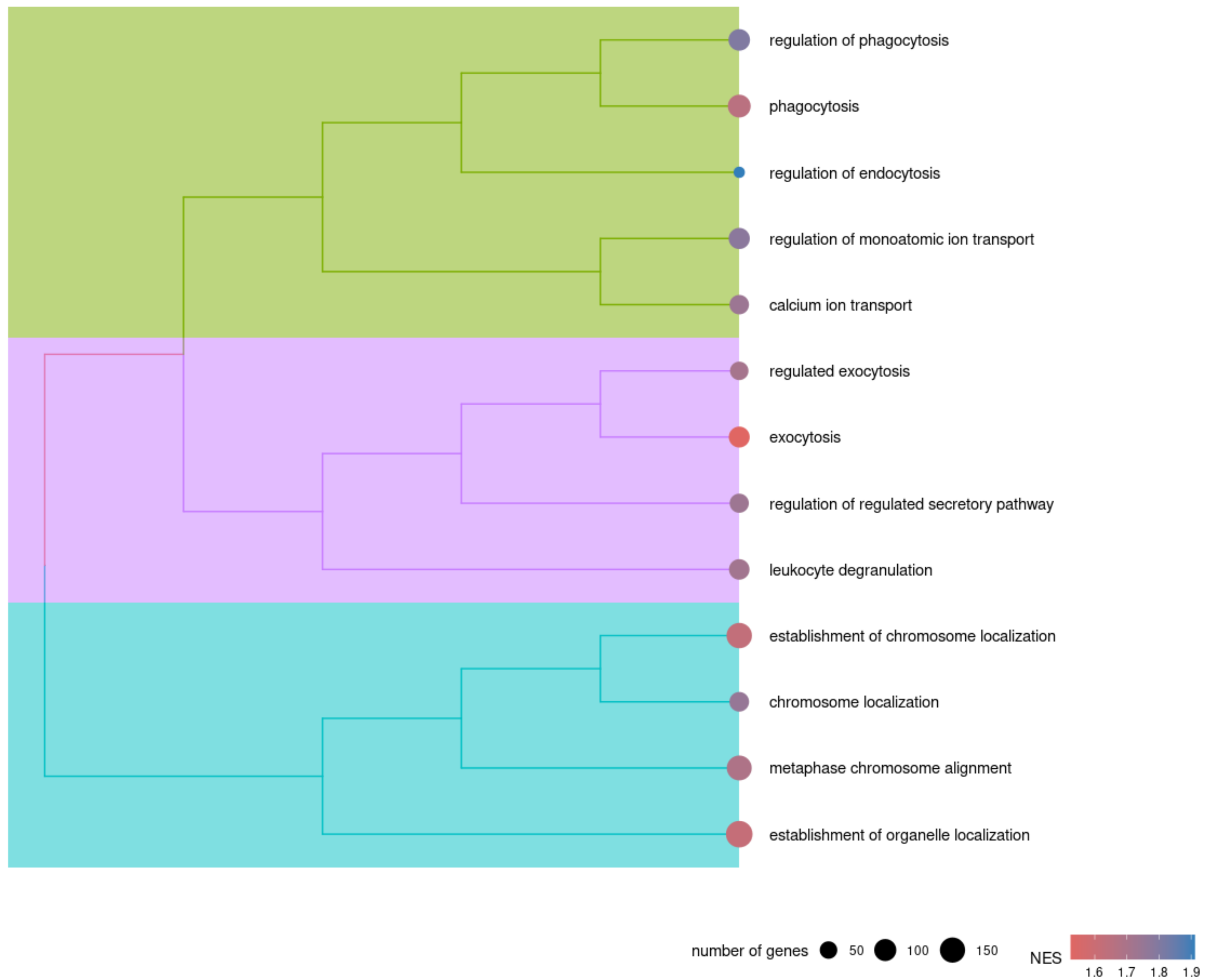

MASH vs Control - Cell adhesion cluster

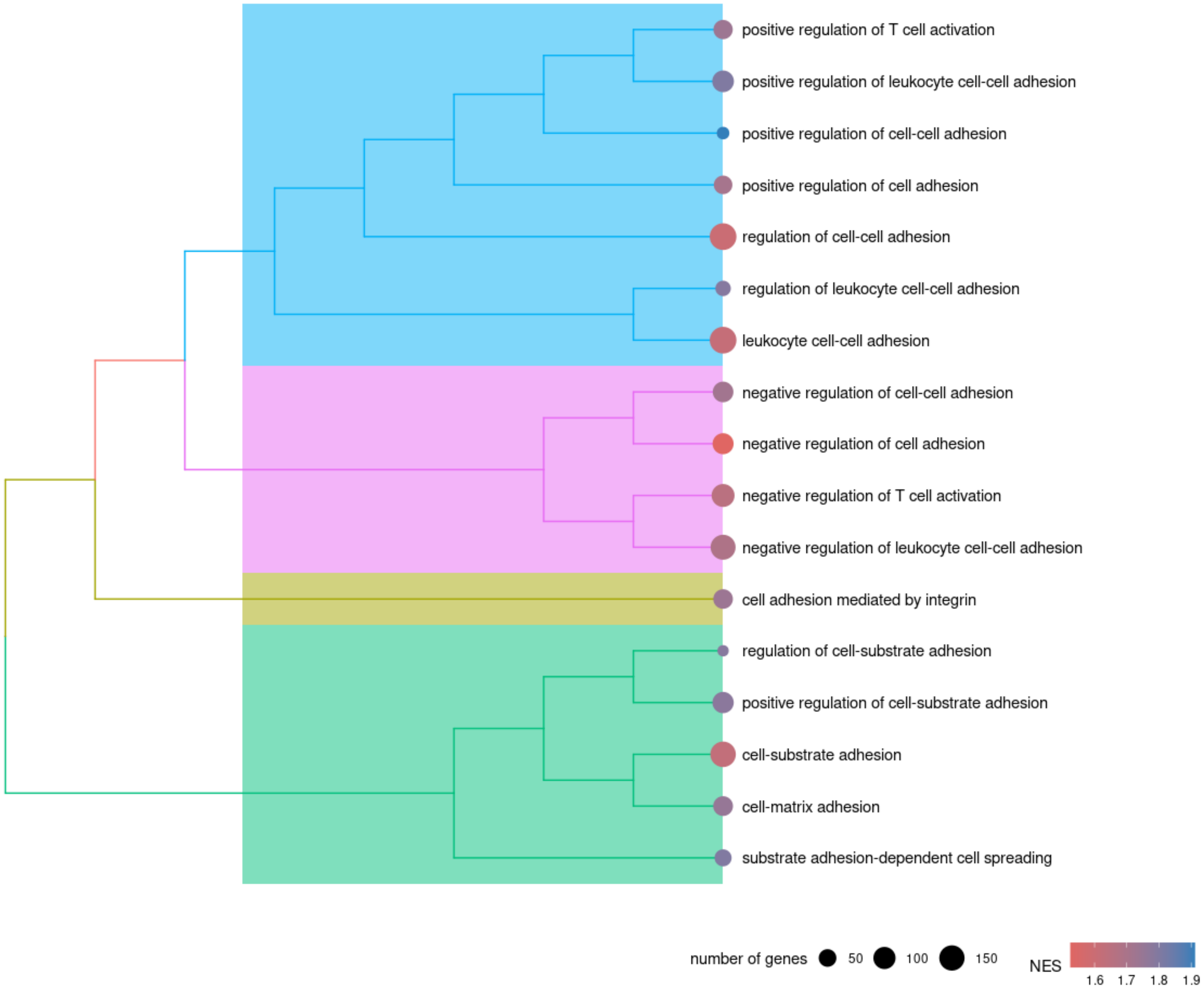

MASH vs Control - Cell division process cluster

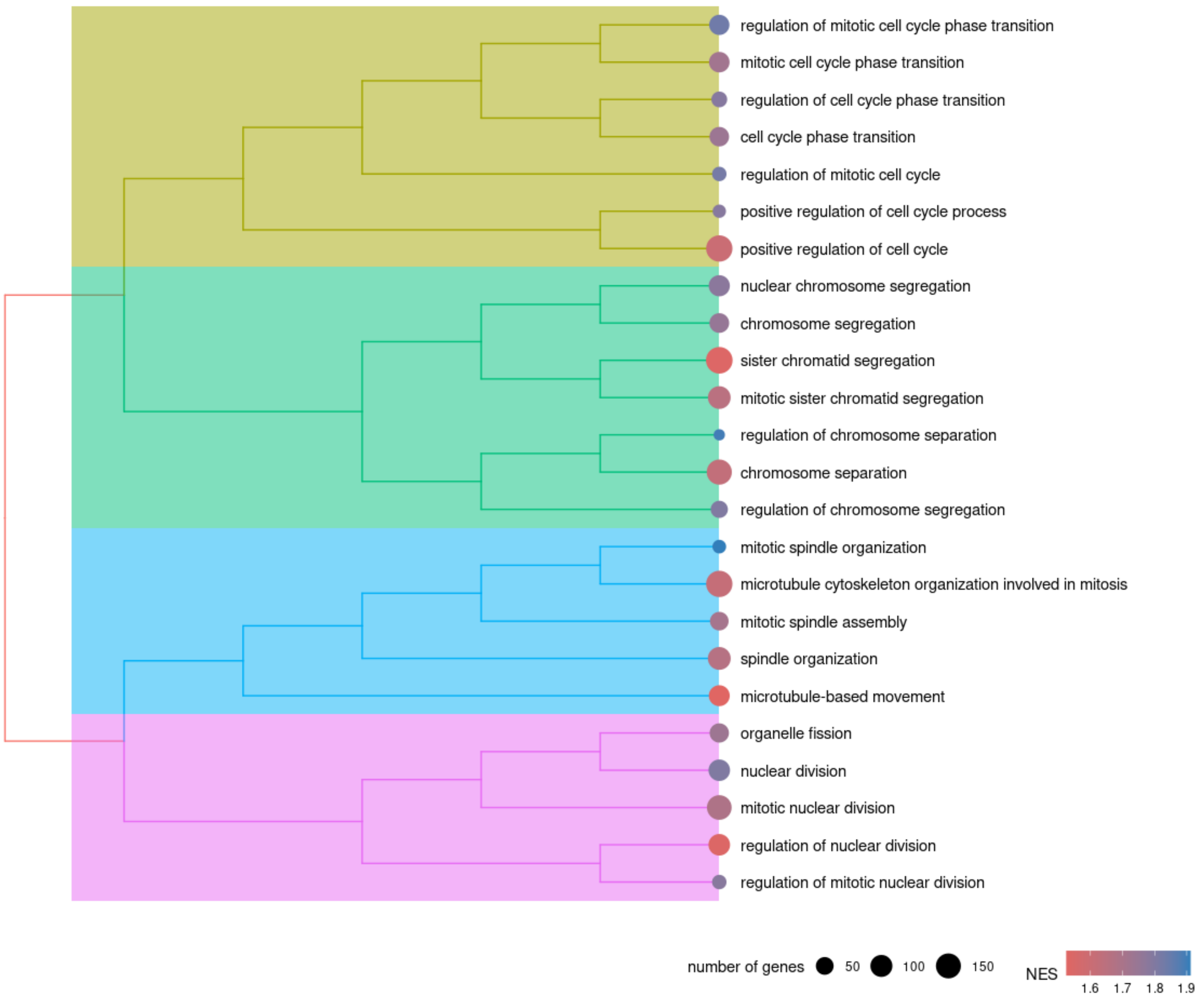

MASH vs Control - TNF & Interleukin production cluster

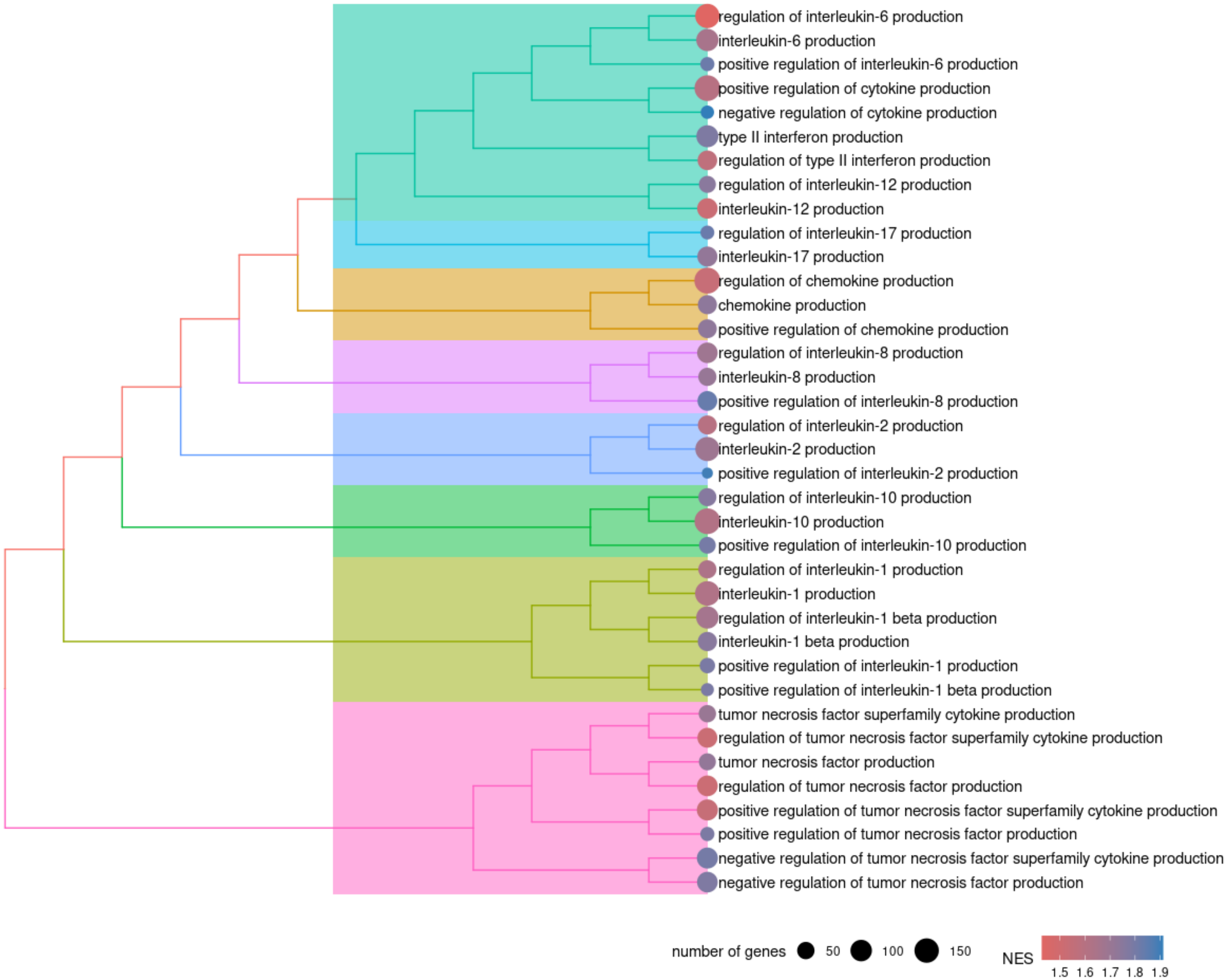

MASH vs Control - Lymphocytes profileration cluster

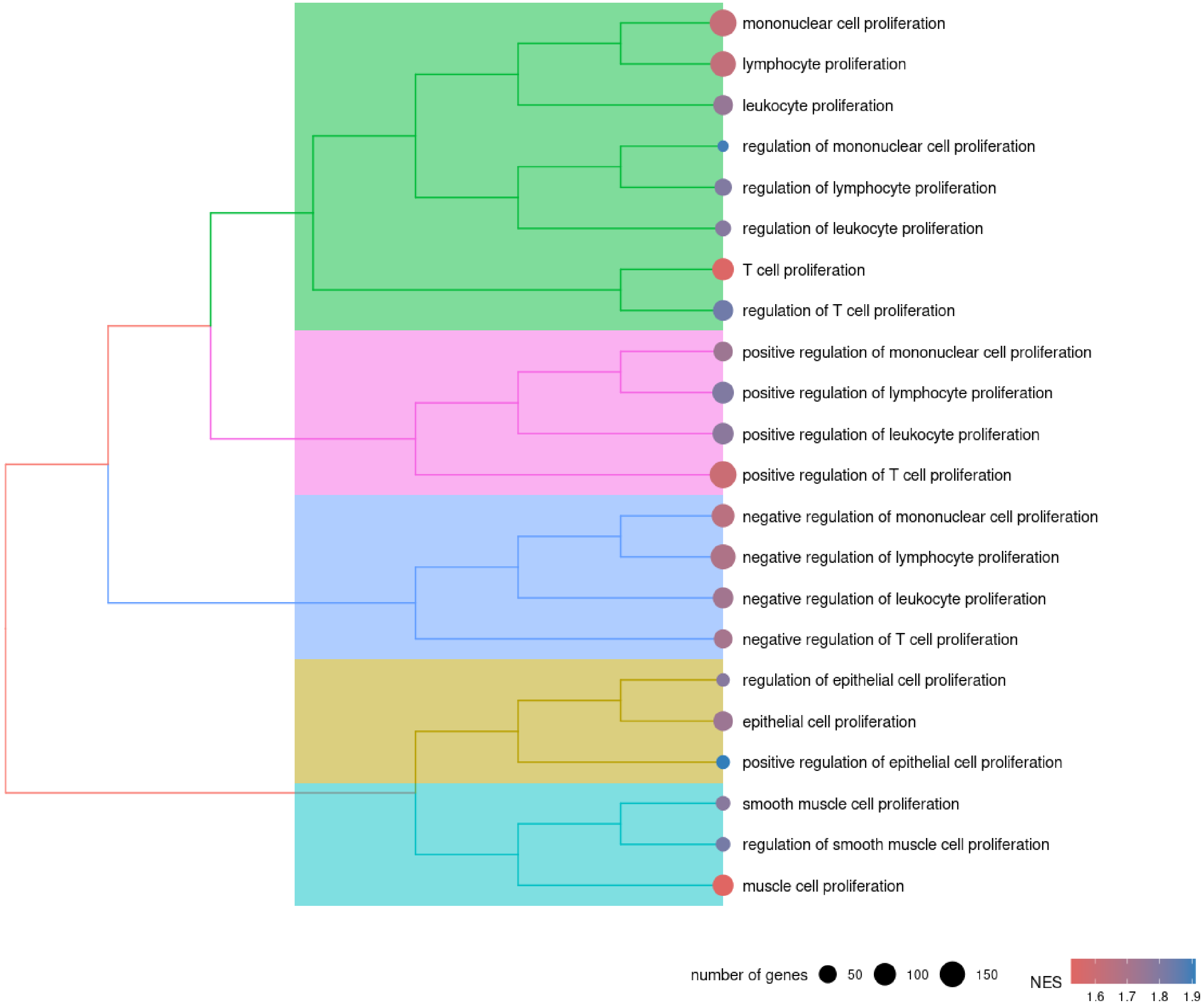

MASH vs Control - Lymphocytes differentation cluster

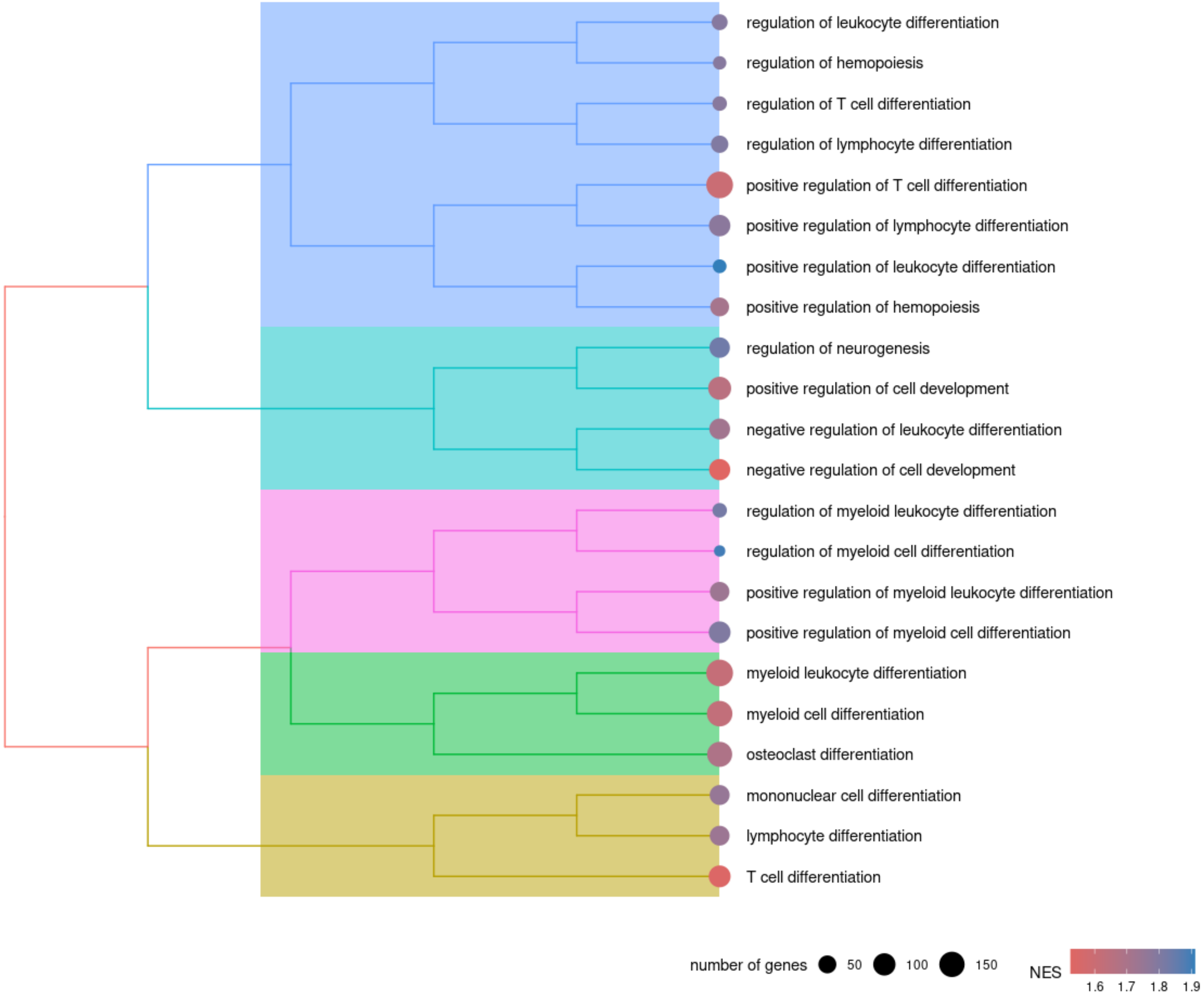

MASH vs MASH + OATD-01 - Extracellular matrix organization cluster

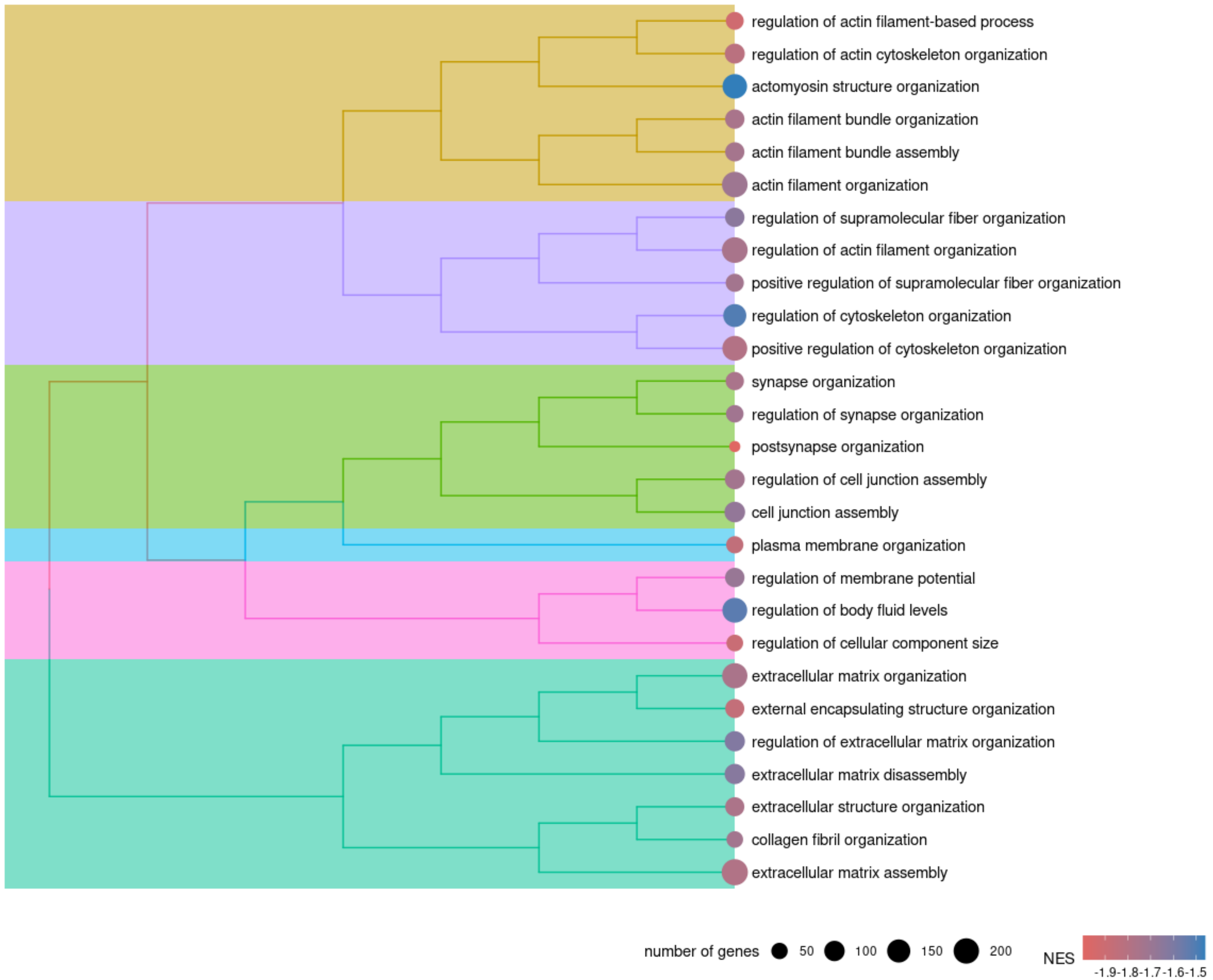

MASH vs MASH + OATD-01 - Lymphocytes activation & differentiation cluster

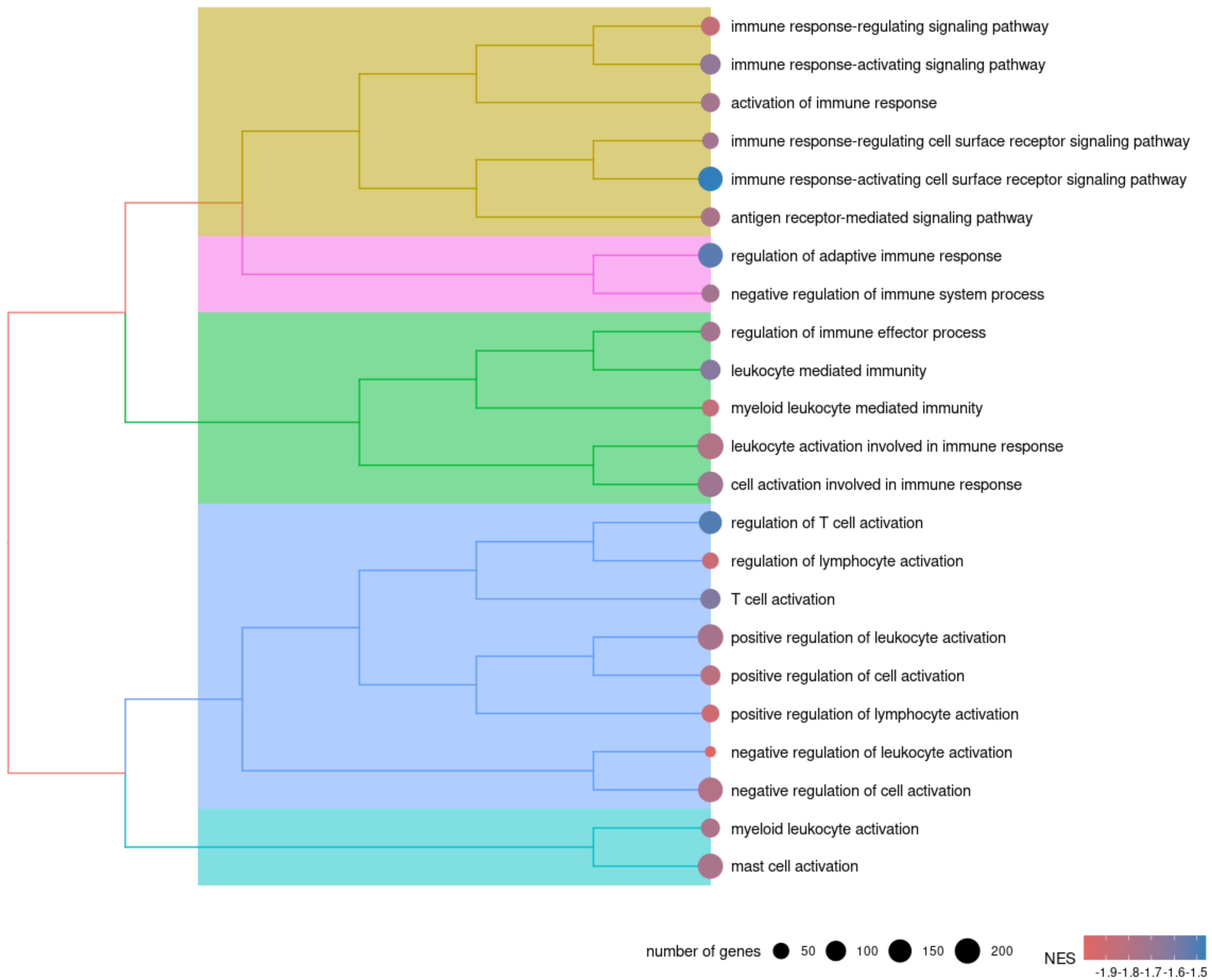

MASH vs MASH + OATD-01 - Wnt signalling pathway cluster

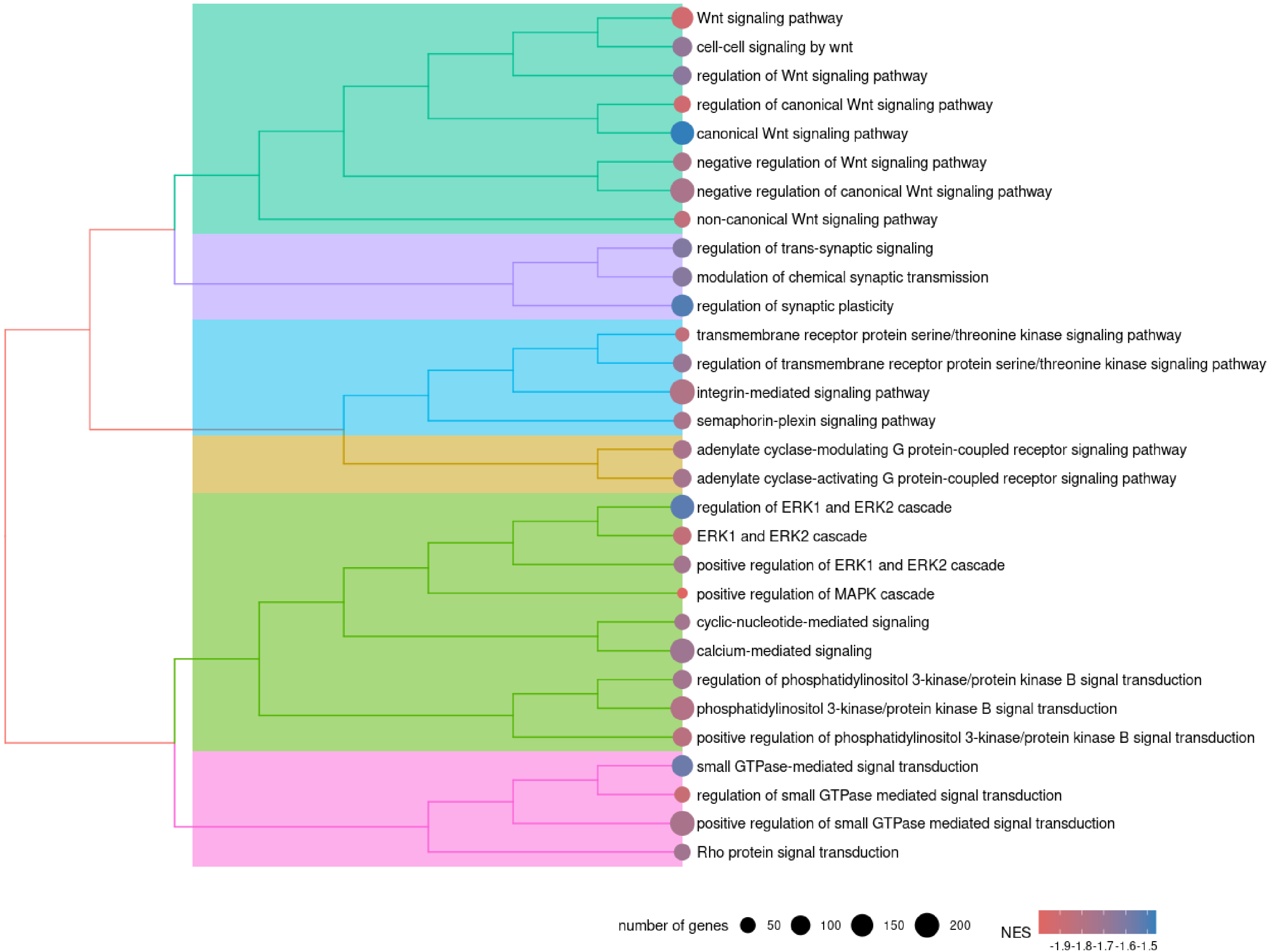

MASH vs MASH + OATD-01 - Myeloid cells activity cluster

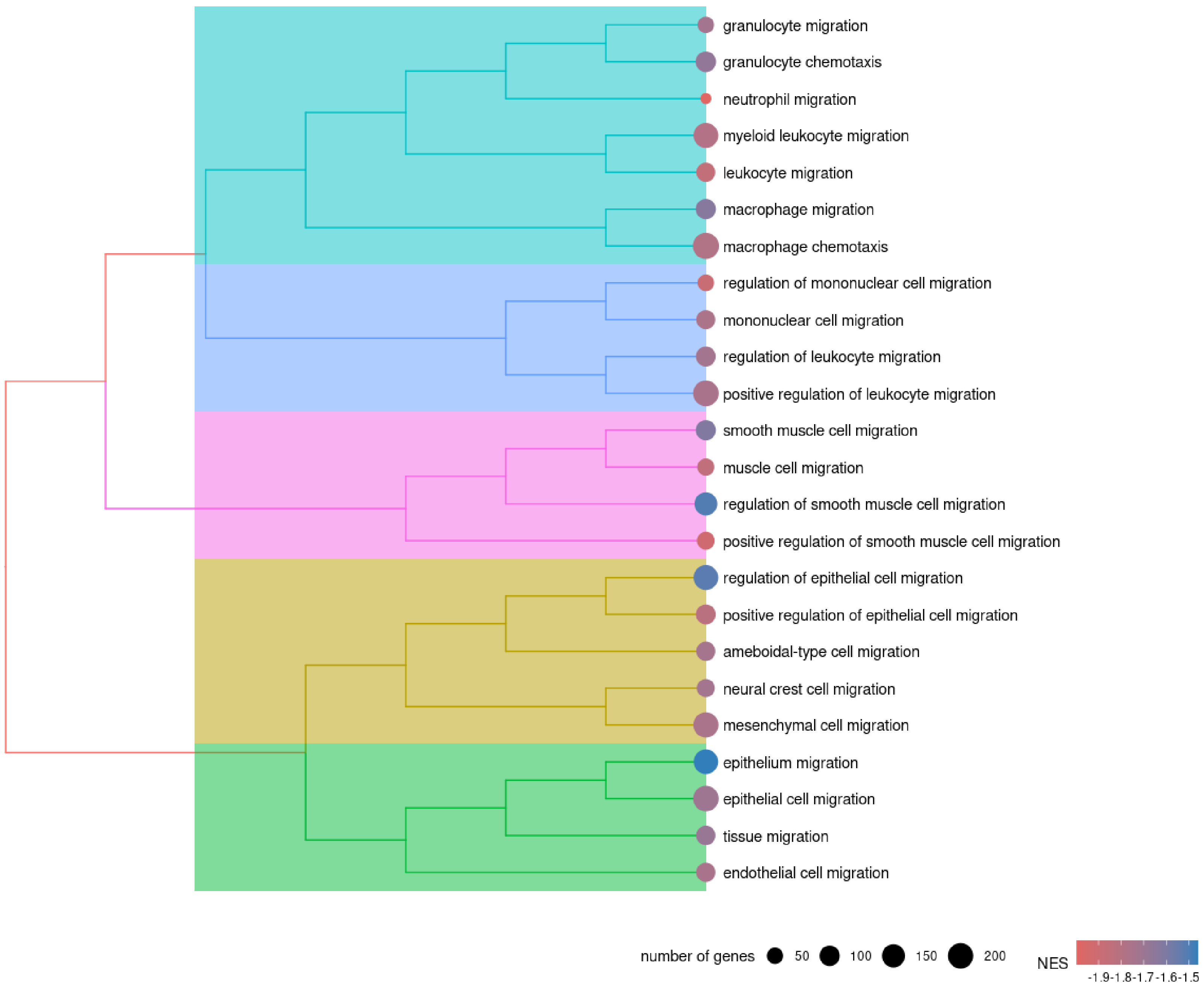

MASH vs MASH + OATD-01 - Mesenchymal / stem cell development cluster

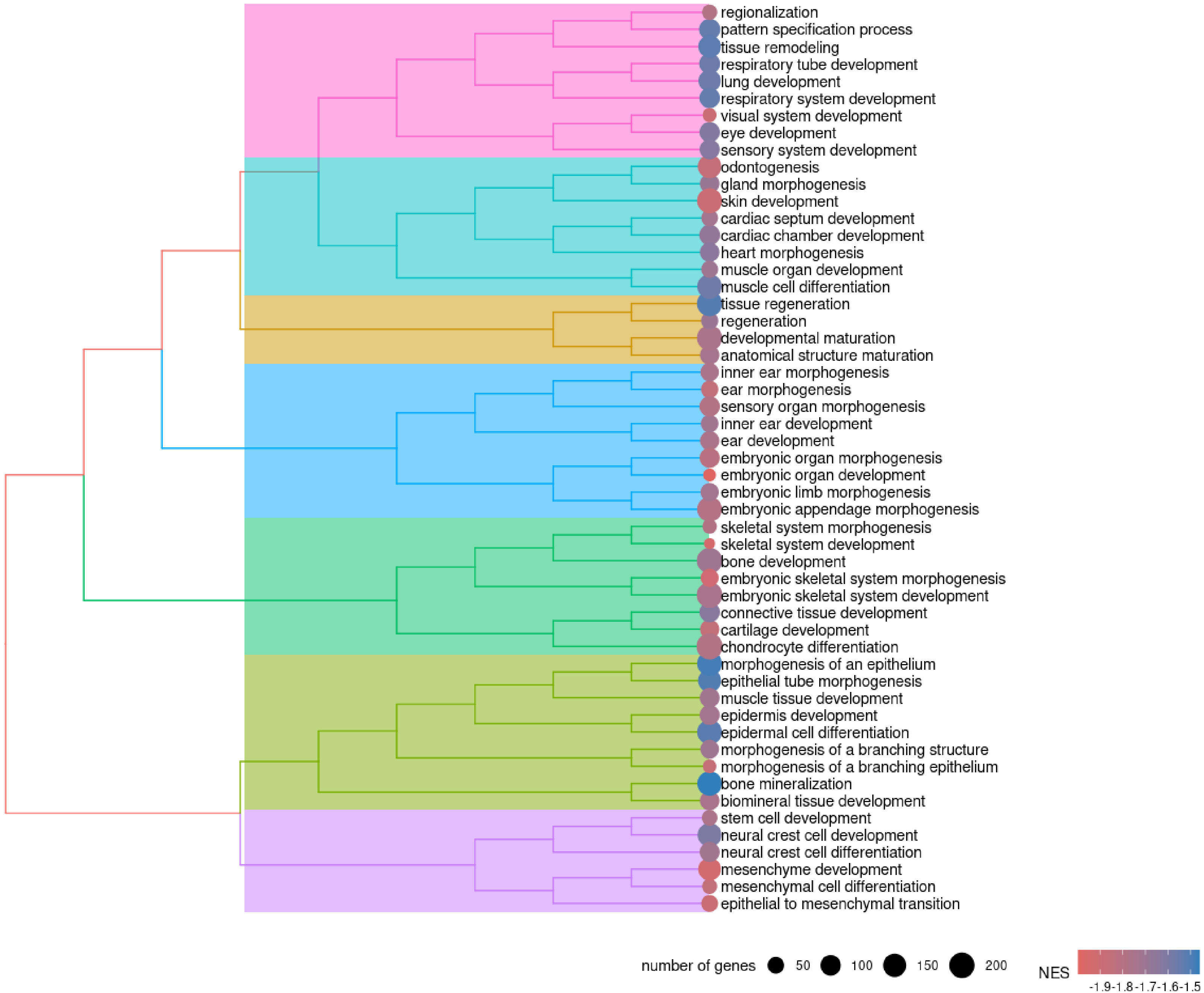

MASH vs MASH + OATD-01 - Regulation of neurogenesis cluster

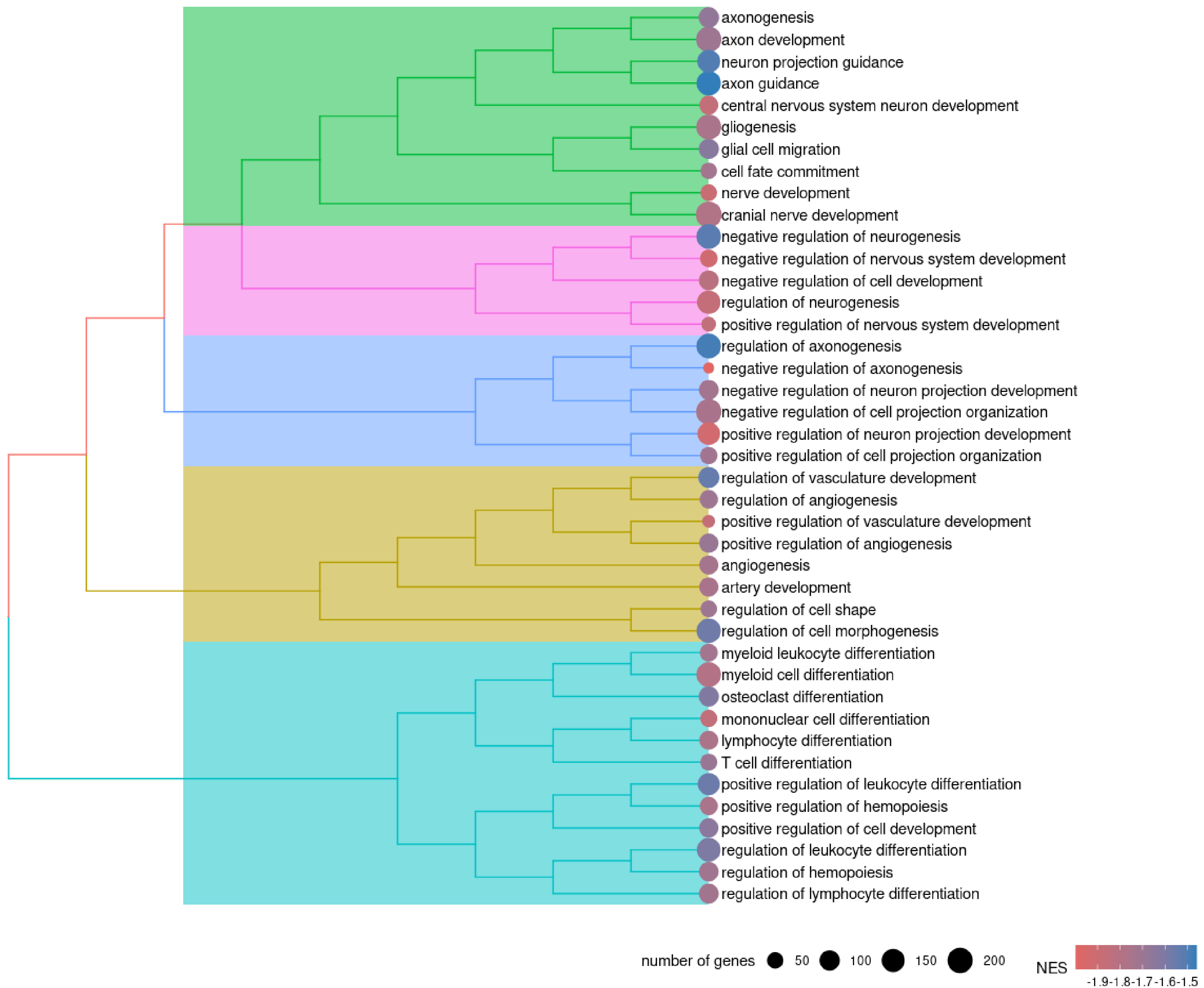

MASH vs MASH + OATD-01 - Regulation of cell growth cluster

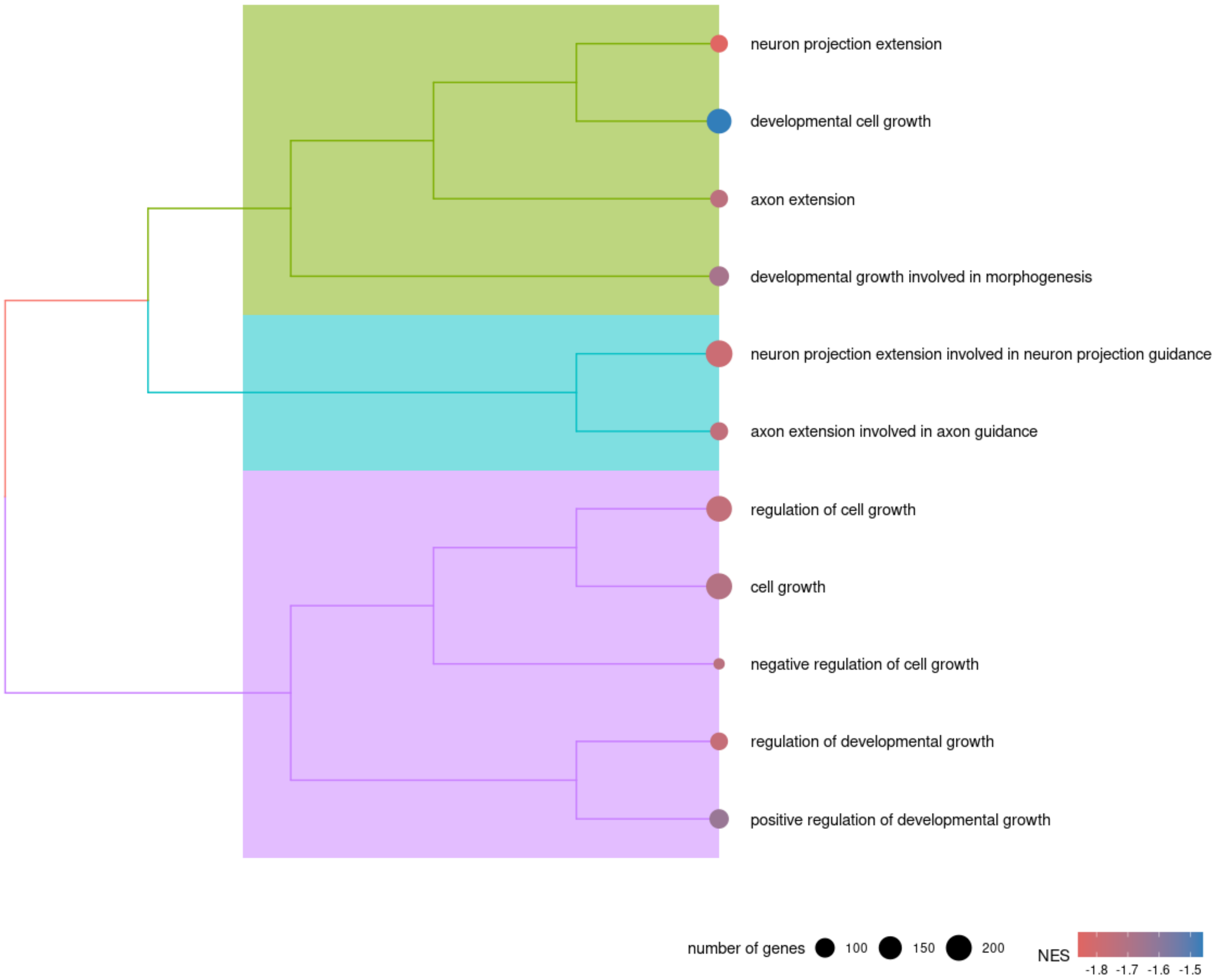

MASH vs MASH + OATD-01 - Blood circulation cluster

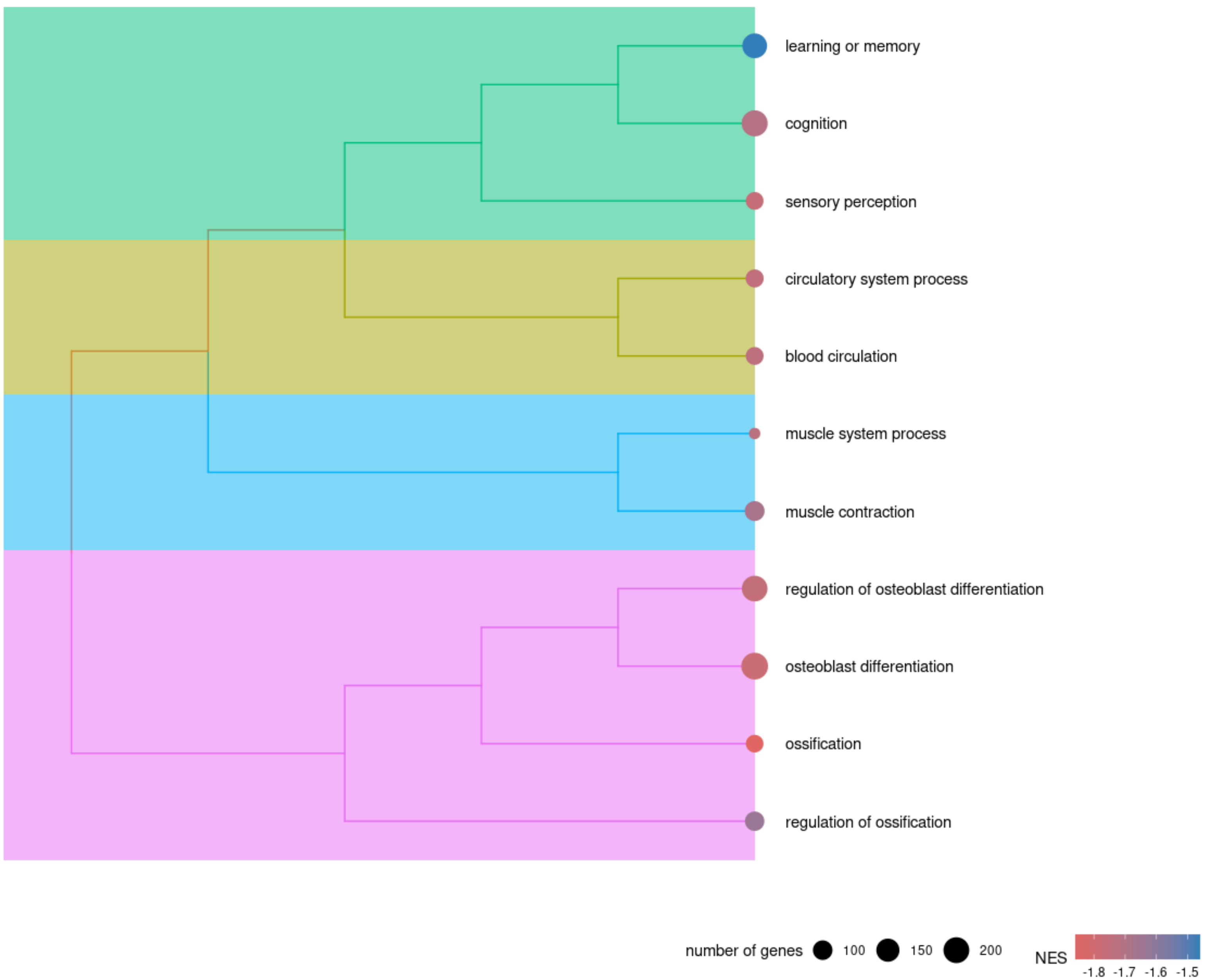

MASH vs MASH + OATD-01 - Regulation of collagen metabolism cluster

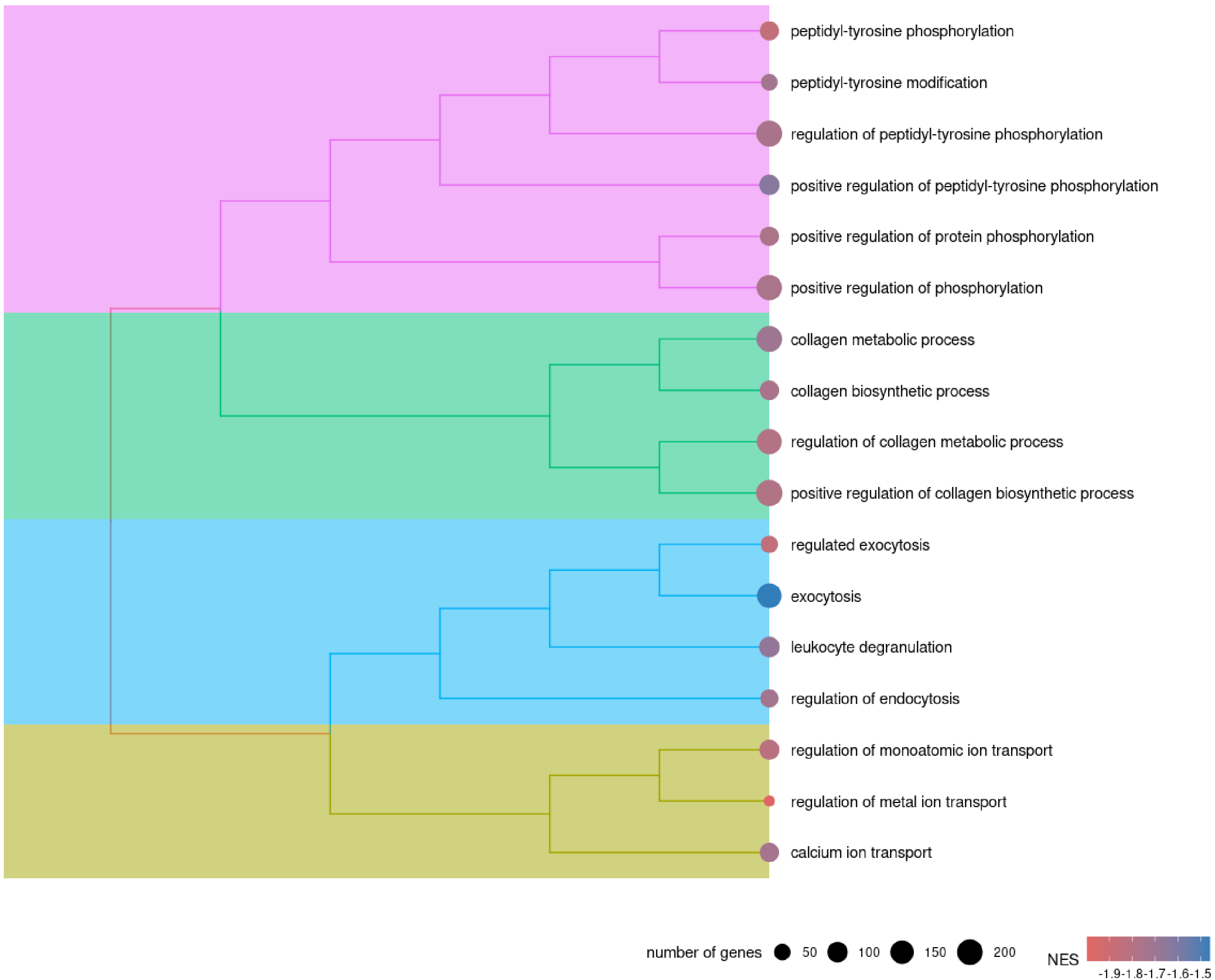

MASH vs MASH + OATD-01 - Cell adhesion cluster

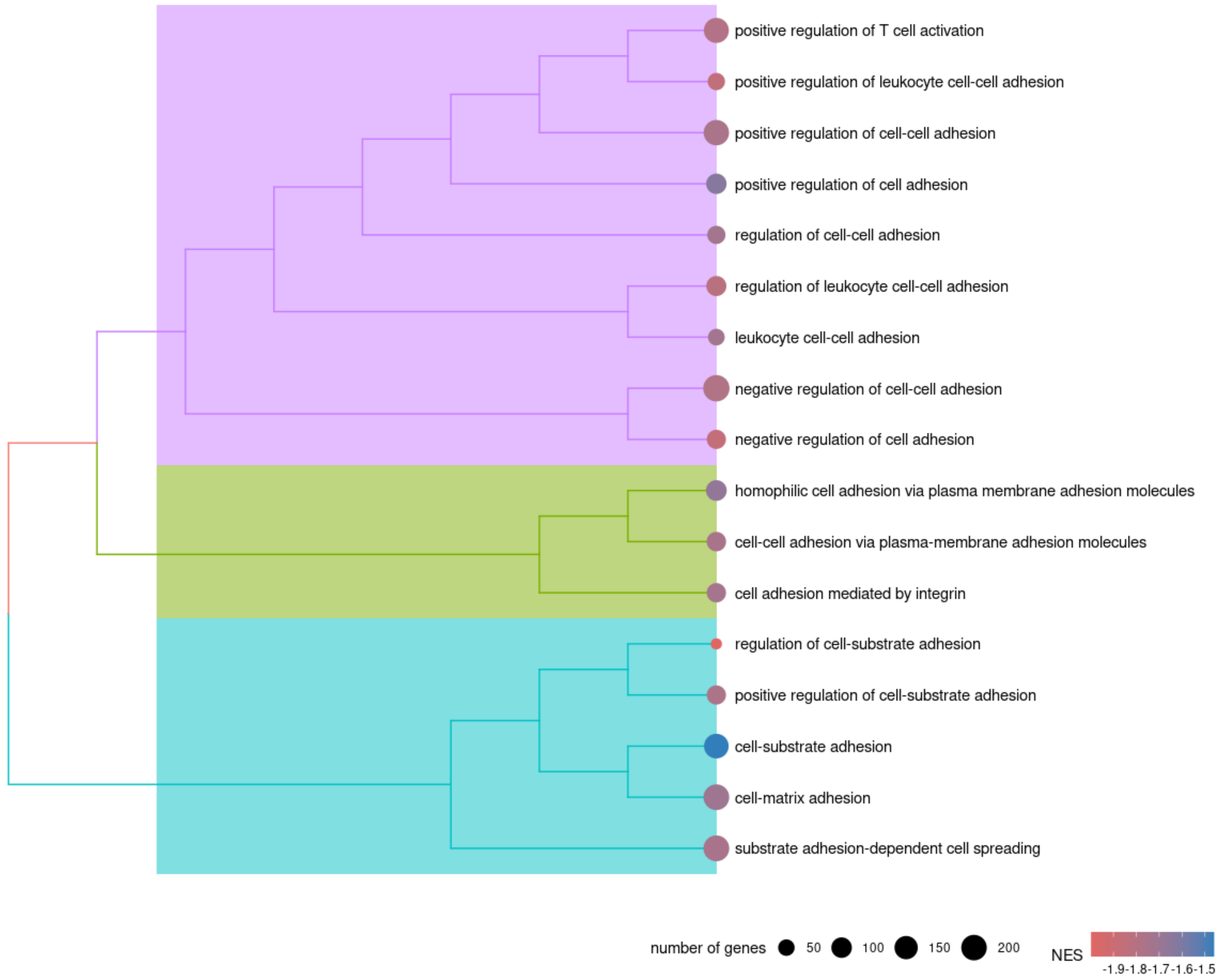

MASH vs MASH + OATD-01 - Chemotaxis cluster

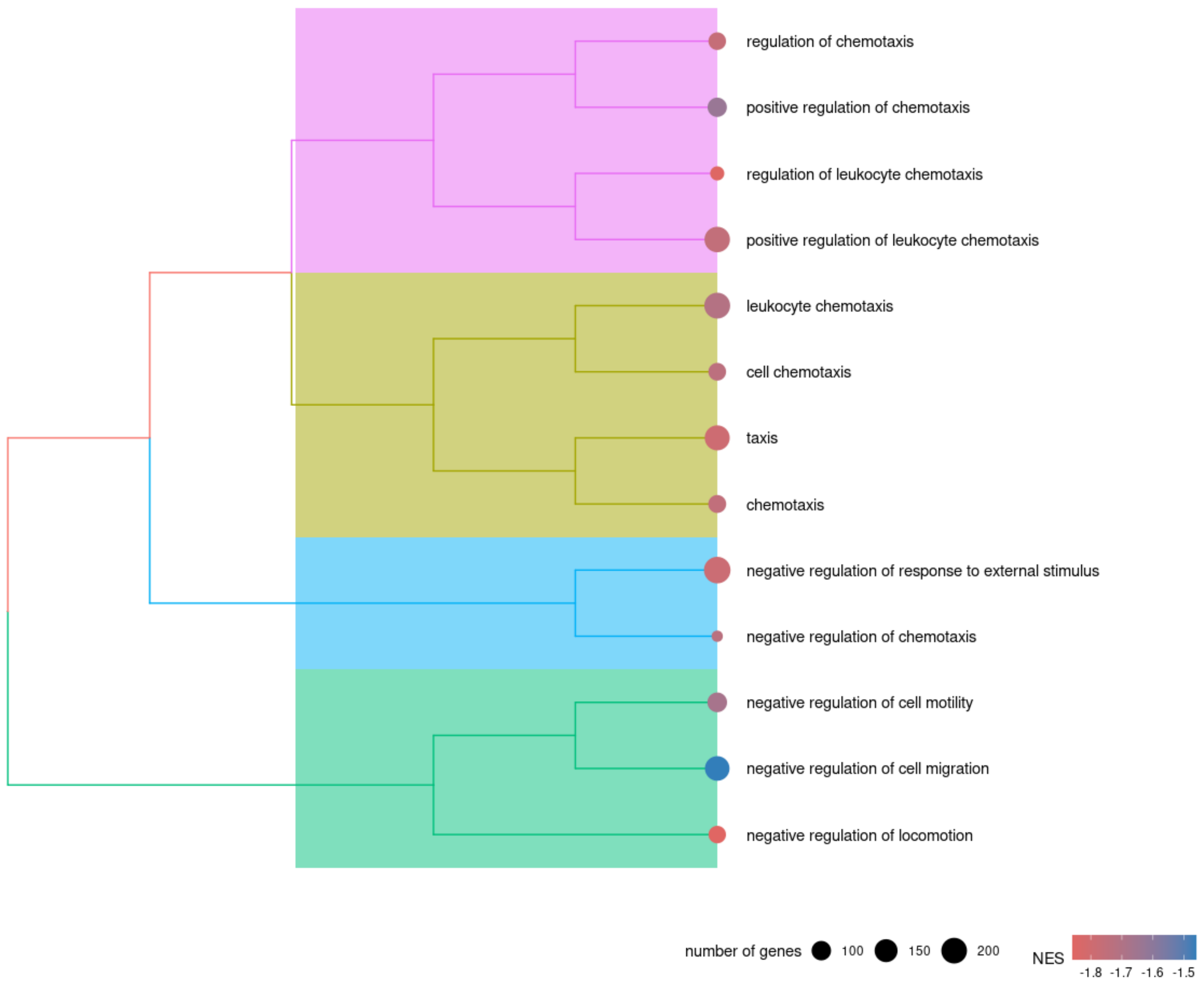

MASH vs MASH + OATD-01 - Epithelial cells development cluster

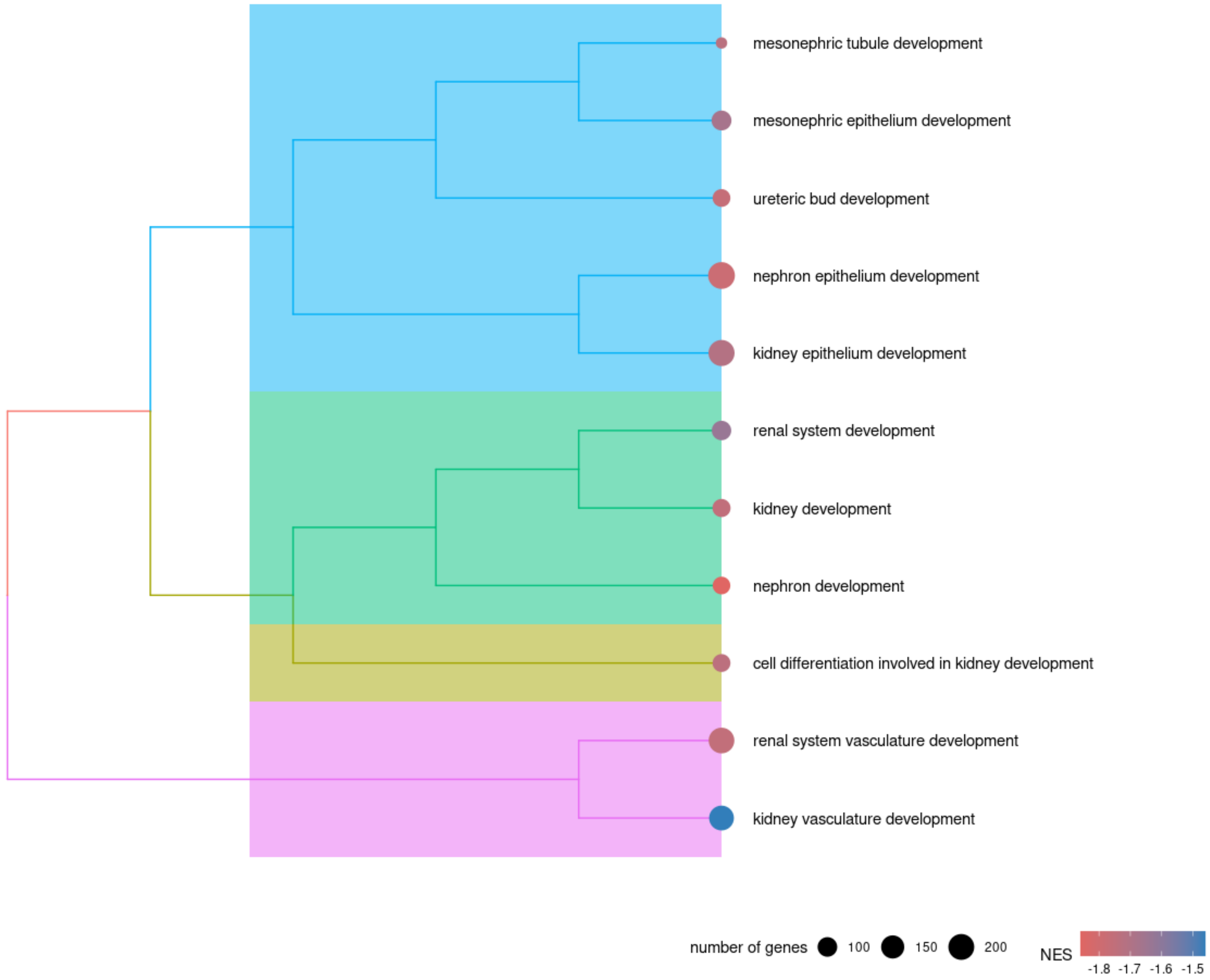

MASH vs MASH + OATD-01 - Cellular response cluster

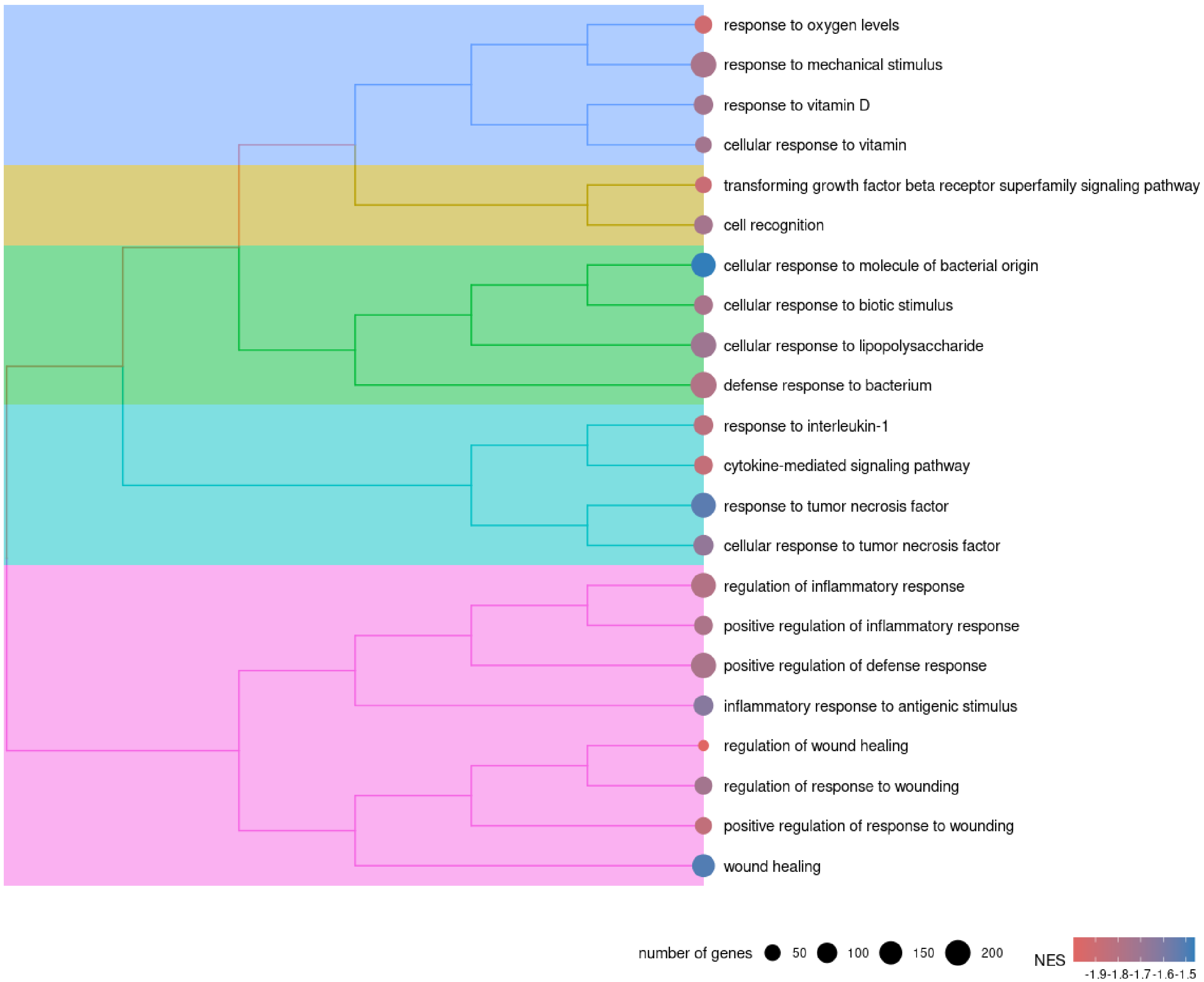

MASH vs MASH + OATD-01 - Cellular response to growth factor cluster

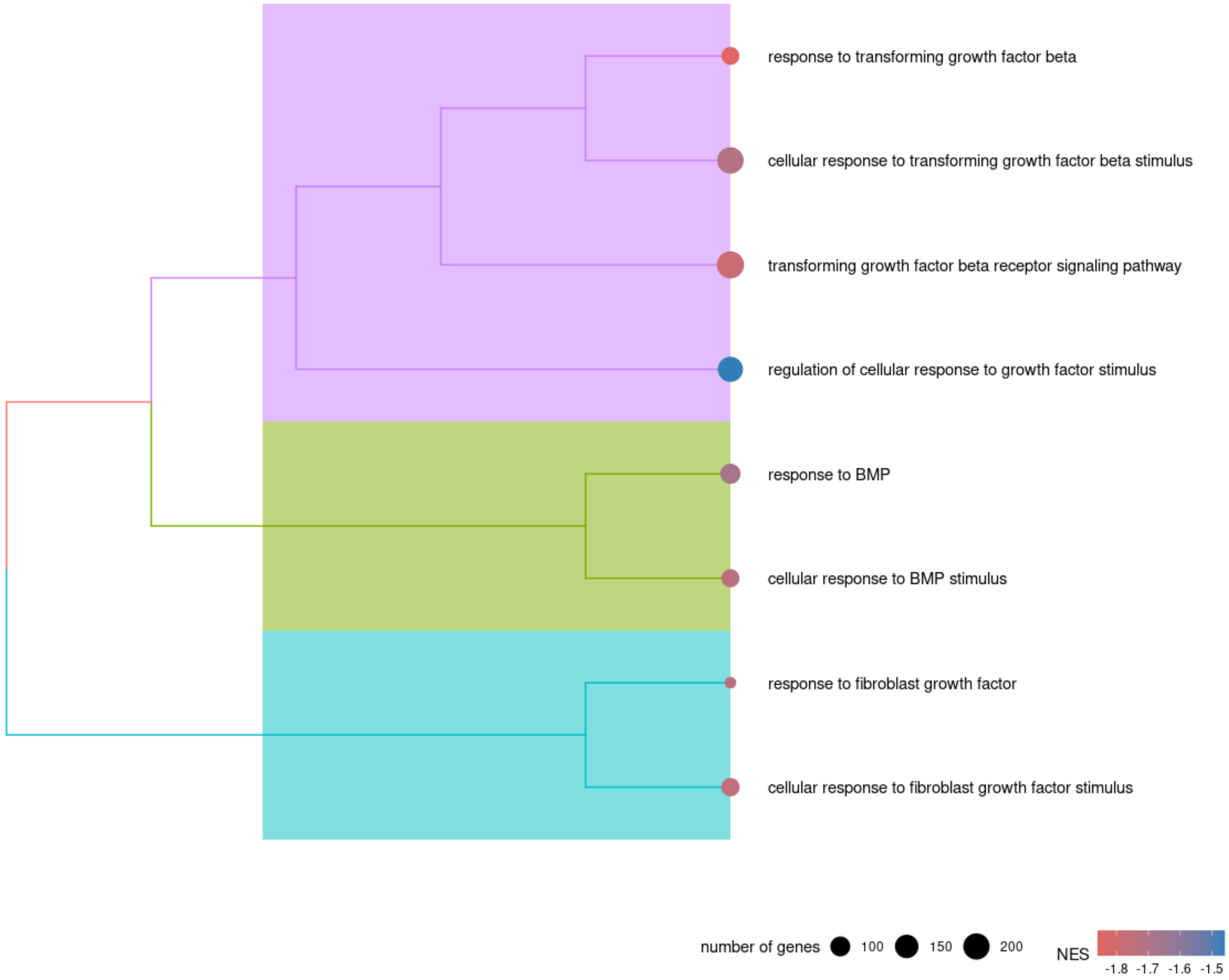

MASH vs MASH + OATD-01 - Lymphocytes profileration cluster

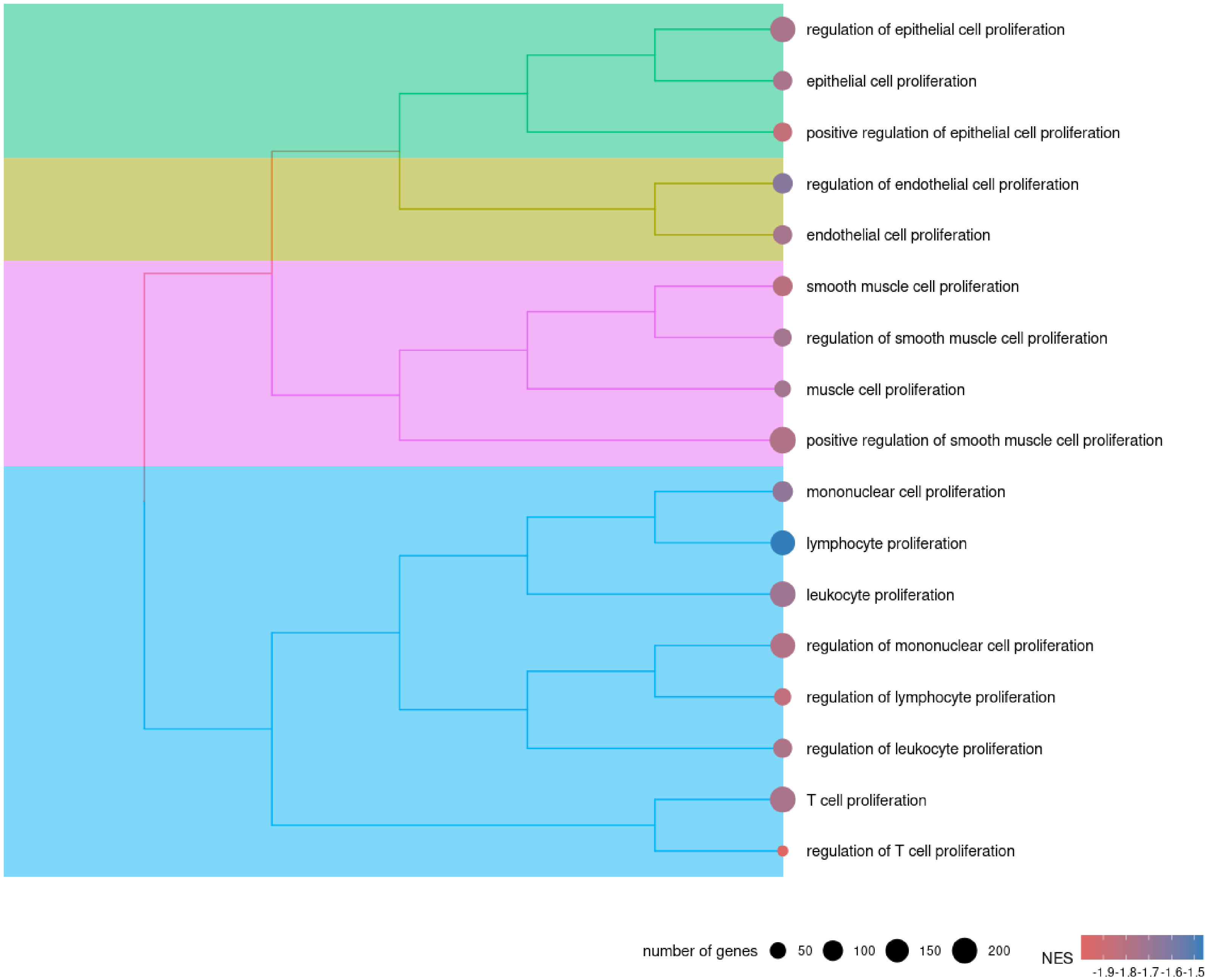

MASH vs MASH + OATD-01 - TNF & Interleukin production cluster

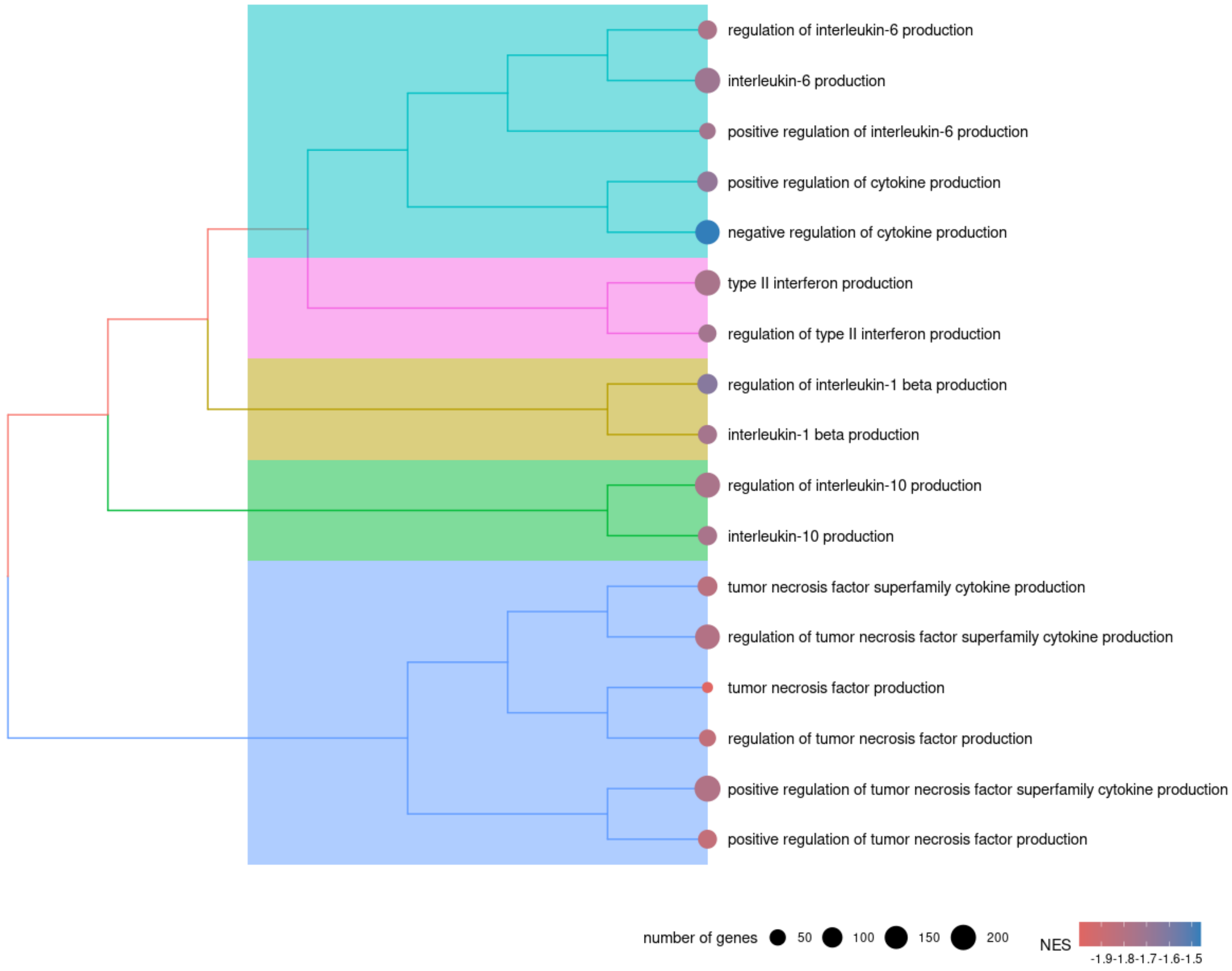

Supplement: Supplementary file 17 [file DataSheet3.pdf]
